# Mathematical Modeling of SARS-CoV-2 Omicron Wave under Vaccination Effects

**DOI:** 10.3390/computation11020036

**Published:** 2023-02-15

**Authors:** Gilberto González-Parra, Abraham J. Arenas

**Affiliations:** 1 Department of Mathematics, New Mexico Tech, New Mexico Institute of Mining and Technology, Socorro, NM 87801, USA; 2 Departamento de Matematicas y Estadistica, Universidad de Cordoba, Monteria 230002, Colombia;

**Keywords:** SARS-CoV-2 variant, Omicron wave, mathematical modeling, vaccination, scenarios, simulations

## Abstract

Over the course of the COVID-19 pandemic millions of deaths and hospitalizations have been reported. Different SARS-CoV-2 variants of concern have been recognized during this pandemic and some of these variants of concern have caused uncertainty and changes in the dynamics. The Omicron variant has caused a large amount of infected cases in the US and worldwide. The average number of deaths during the Omicron wave toll increased in comparison with previous SARS-CoV-2 waves. We studied the Omicron wave by using a highly nonlinear mathematical model for the COVID-19 pandemic. The novel model includes individuals who are vaccinated and asymptomatic, which influences the dynamics of SARS-CoV-2. Moreover, the model considers the waning of the immunity and efficacy of the vaccine against the Omicron strain. This study uses the facts that the Omicron strain has a higher transmissibility than the previous circulating SARS-CoV-2 strain but is less deadly. Preliminary studies have found that Omicron has a lower case fatality rate compared to previous circulating SARS-CoV-2 strains. The simulation results show that even if the Omicron strain is less deadly it might cause more deaths, hospitalizations and infections. We provide a variety of scenarios that help to obtain insight about the Omicron wave and its consequences. The proposed mathematical model, in conjunction with the simulations, provides an explanation for a large Omicron wave under various conditions related to vaccines and transmissibility. These results provide an awareness that new SARS-CoV-2 variants can cause more deaths even if their fatality rate is lower.

## 1. Introduction

Over the course of the COVID-19 pandemic, at least 671 million confirmed cases and 6.83 million deaths have been reported (December 2022) [1]. These reported numbers are in the lower bounds, since there are asymptomatic and under-reported cases [2–7]. During 2019, 2020, 2021 and 2022, different strains of the SARS-CoV-2 virus have been found [8–13]. These strains have different characteristics related to contagiousness and severity. Thus, some SARS-CoV-2 variants affect the count of infected cases, hospitalizations and deaths [14,15]. Vaccination programs against SARS-CoV-2 started at the very end of 2019 and the beginning of 2020 in some countries [16–21]. For the year 2022, many countries have already implemented vaccination programs and some countries have also implemented booster programs [4,22–24]. The evolution of SARS-CoV-2 is affected by various factors that are difficult to quantify [25–28]. For instance, social behavior and vaccination status are major factors that influence the COVID-19 pandemic [27,29–37]. New SARS-CoV-2 strains also play a major role in the evolution of the COVID-19 pandemic and have generated different spatial-temporal waves in different countries [12,38–44]. These waves are mainly the product of different contagiousness of new SARS-CoV-2 strains and public health interventions.

The Omicron variant caused a new wave during 2022. The count of cases has been very large and has exceeded previews waves. Omicron was first detected in South Africa and Botswana in early November 2021, but using retrospective testing, it was found that Omicron was also present in England, Nigeria and the United States during November of 2021 [45–47]. Omicron has more than fifty mutations in comparison with the original circulating SARS-CoV-2 [46]. The Omicron strain carries an unusually high number of mutations, suggesting potential immune evasion [22]. A near-complete lack of neutralizing activity has been reported against Omicron in polyclonal sera from individuals vaccinated with two doses of the BNT162b2 COVID-19 vaccine and from convalescent individuals, as well as resistance to different monoclonal antibodies in clinical use [22]. In [48], results suggest that two doses of COVID-19 vaccines only offer modest protection against symptomatic Omicron infection. In [24], the authors showed that Omicron exhibits significant immune evasion compared to other strains. In addition, they found that the Omicron spike exhibits reduced receptor binding and cell–cell fusion, but increased cell-to-cell transmission [24].

Despite the fact that the Omicron strain has lower severity, it has caused a large number of hospitalizations and the average daily number of deaths has been substantial [49]. Some studies have reported a lower rate of hospitalization for the Omicron strain compared with infections caused by the Delta strain [50]. It has been found that booster vaccination and vaccination of individuals with a history of SARS-CoV-2 infection generated lower antibody titers than those against the Delta strain [51,52].

One important aspect for studies predicting health outcomes related to this pandemic is how deadly each of the SARS-CoV-2 strains are. There are two main ways to compute how deadly a disease is. The first is the infection–fatality ratio (IFR), which is given by the ratio of deaths to all infected individuals. The second is computing the case fatality ratio (CFR), which is given by the ratio of deaths to confirmed cases. Estimating the IFR is complex, since it requires knowing the total number of infected cases. Some studies have estimated the CFR as being from less than 0.1% to over 25% [53]. For COVID-19, the true level of transmission is frequently underestimated because a substantial proportion of people with the infection are undetected, either because they are asymptomatic or are not reported [53–55]. In places where testing is extensive, the estimation of CFR is more robust [56]. Another aspect that affects health outcomes is the immunity level of the population which is related to the herd immunity. The increase in population immunity makes it more difficult to compare Omicron’s severity with previous circulating SARS-CoV-2 strains, since previous exposure to SARS-CoV-2 strains is expected to prevent to some extent severe outcomes from subsequent infection [57].

The main objective here is to obtain insight into the impact of the Omicron strain. In particular, our aim is to propose a mathematical approach that helps to provide an explanation of the large Omicron wave and the great number of deaths during this wave despite its lower fatality rate. We propose a mathematical modeling framework to study the Omicron wave and attain some additional insight into its evolution. Mathematical models are fruitful and have been used to investigate a variety of scenarios related to the behavior of the COVID-19 disease [6,58–74]. These models are used to study the impact of a variety of health interventions on epidemics. With in silico simulations of the mathematical models we can produce a variety of outcomes that are difficult to foresee due to the nonlinearity and complexity of the epidemics [75–77]. In addition, in some cases the mathematical analysis permits us to determine under what conditions the disease can disappear. Previous studies have investigated the dynamics of the COVID-19 pandemic under two SARS-CoV-2 variants, but some of them did not include vaccination and waning since they were designed for the early pandemic [78–85]. Recently, some researchers have studied the Omicron wave dynamics [86–89]. In [88], the authors analyzed a second wave of COVID-19 and in particular on the Omicron variant pandemic data in India. In [86], a stochastic and second-order model is proposed to deal with the Omicron wave. A mathematical model considering age structure, vaccine, antiviral treatment and influx of the Omicron variant in Korea was developed in [87]. The authors in [58] proposed a fractal–fractional age-structure model for the omicron SARS-CoV-2 variant and considered two age groups. They found that there is a high infection and recovery rate of the Omicron SARS-CoV-2 variant infection among the population under 50. In [90], a generalized SIR model was used to simulate and predict the dynamics of Omicron waves in Ukraine and in the whole world. Mathematical models also have bee used for within-host dynamics for SARS-CoV-2 and in particular the Omicron variant [91,92]

Over this pandemic, many SARS-CoV-2 strains have appeared and these have different characteristics [11,93–97]. Previous models have been used to investigate the influence of new SARS-CoV-2 strains that have a higher probability of transmission [6,38,79,80,83,98–101]. In particular, some interesting studies have considered the mathematical modeling of new SARS-CoV-2 strains and at the same time imperfect vaccination or waning [83,99,101]. Furthermore, some mathematical models have been proposed for studying SARS-CoV-2 waves [40,102,103]. The models have different underlying assumptions and, as any mathematical model of an epidemic, they have advantages and limitations. A variety of work has been carried out considering continuous and discrete models that have included vaccinated subpopulations where people have less probability to get infected, proliferate the virus, or die [78,81,101,104–108].

In this study, we build a mathematical model for the Omicron wave situation. Individuals who are asymptomatic and vaccinated are included in the model since they influence the evolution of the Omicron wave [109–115]. In this study, we use the fact that the Omicron strain has a higher transmissibility than the previously circulating SARS-CoV-2 strains and that the vaccine efficacy is lower for the Omicron strain [22,48]. In addition, we take into account that preliminary studies have found that Omicron has a lower case fatality rate compared to previous circulating SARS-CoV-2 strains. We perform in silico simulations with a variety of scenarios to attain insight into the Omicron wave, its potential consequences and to explain the Omicron wave situation. In this study, we perform a brief stability analysis of the developed model and we also identify the basic reproduction number ${ℛ}_{0}$ despite the fact that the in silico simulations are aimed more toward shorter dynamics [116,117]. The reproduction number ${ℛ}_{0}$ is strongly connected to the effective reproduction number ${ℛ}_{t}$, and therefore is useful in obtaining insight into the behavior of epidemics and pandemics. The motivation of this work is to provide additional knowledge-based support to health authorities and the population in general. Scientific studies that bring awareness of health issues are important to public health despite sometimes the scientific tools used not being very complex [118]. In summary, we propose a mathematical approach to provide an explanation of a large Omicron wave arising under various conditions as a function of vaccination status and transmissibility. These results provide awareness that new SARS-CoV-2 variants can cause more deaths even if their fatality rates are lower.

There are some certain previous studies and mathematical models related to the Omicron wave [119–121]. In [121], the authors implemented a stochastic, discrete-time-, individual-based transmission model of SARS-CoV-2 infection and COVID-19 disease. The model considers an age-structured, small-world network. Using sensitivity analysis due to many uncertainties they show that a new SARS-CoV-2 variant dominance is primarily driven by its infectivity, which does not necessarily lead to an increased public health burden. In [119], the authors used a model fitted to more than 2 years of epidemiological data from England to project potential dynamics of SARS-CoV-2 infections and deaths in England to December 2022. They considered several key uncertainties including behavioral changes and waning immunity. They concluded that for the particular case of England and under the assumption that no new variants emerge, SARS-CoV-2 transmission is expected to decline. The authors concluded that the projections depend largely on assumptions around waning immunity, social behavior and seasonality. Other interesting work related to Omicron waves is presented in [120]. In this work, a generalized SEIR model assuming gamma-distributed incubation and infectious periods is presented. The model includes susceptibility to Omicron. Their results suggest that even in those regions where the Delta variant is controlled before the beginning of the Omicron wave a significant Omicron wave can be expected. It is important to remark that for the particular case of England the Omicron wave was smaller than the Delta wave. In our paper, we provide additional insight regarding the Omicron wave.

This paper is organized as follows: In Section 2, we build the mathematical model for the Omicron wave dynamics. Section 3 is devoted to the stability analysis of the model. In Section 4, the numerical simulation results regarding the Omicron wave are presented, and the final section is devoted to discussion and conclusions.

## 2. Mathematical Model for the Omicron Wave Dynamics

We constructed a mathematical model that relies on nonlinear differential equations. The model includes the Omicron strain and one previous circulating strain of SARS-CoV-2. The mathematical model uses the fact that Omicron is more contagious than the previously circulating SARS-CoV-2 strain. The constructed model also encompass people who are vaccinated and asymptomatic. Moreover, the model assumes the waning of immunity for vaccinated and recovered individuals. All these are major components of the constructed model and a novelty in comparison with other models. The developed model assumes that the pre-existent circulating SARS-CoV-2 strain(s) has (have) lower contagiousness than the Omicron strain. The constructed model can be extended to other circulating SARS-CoV-2 strains if similar conditions hold.

**Figure 1.**
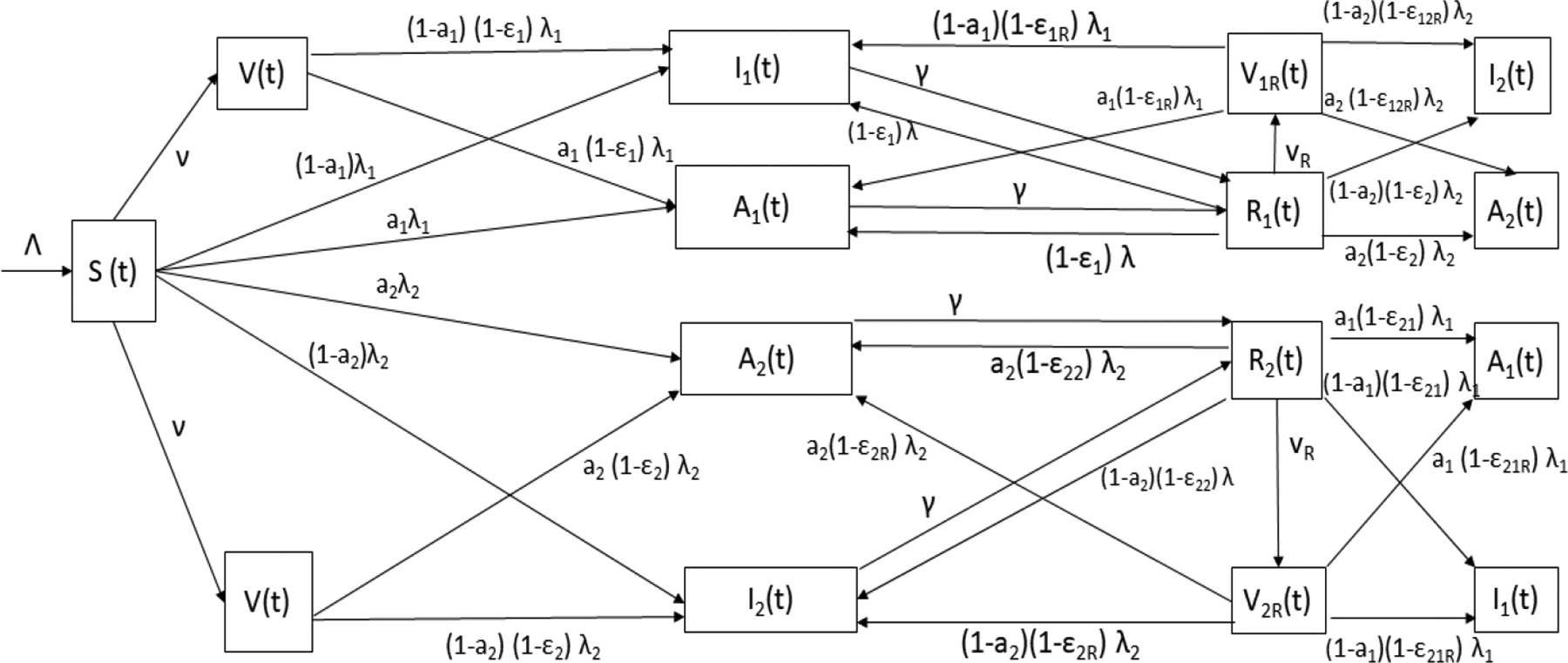
Diagram of the mathematical model (2) with classes and relevant parameters.

The model encompass individuals in the susceptible $\left(S_{i}(t)\right)$, symptomatic $\left(I_{i}(t)\right)$, asymptomatic $\left(A_{i}(t)\right)$ and recovered $\left(R_{i}(t)\right)$ groups for each SARS-CoV-2 strain. In addition, the model comprise three type of subclasses for vaccinated individuals. The first is when susceptible individuals are vaccinated V(t), the second when individuals who have recovered from strain 1 get vaccinated $V_{1R}$, and the last arises when individuals who have recovered from strain 2 get vaccinated $V_{2R}$. The individuals in the last two subpopulations have stronger immunity and protection against the SARS-CoV-2, as immunology studies have suggested [57]. The model is depicted in Figure 1.

The flow of individuals from one subpopulation to another depends on the individual COVID-19 disease status. The model bears in mind partial cross-immunity against the other SARS-CoV-2 strain due to the adaptive immune response [75,122–124]. A susceptible individual can get infected with either strain and progress to the symptomatic classes (with either the previously circulating strain or Omicron) or to the asymptomatic classes ($A_{1}(t)$ or $A_{2}(t)$). The symptomatic and asymptomatic individuals stay in the infectious stage for a certain time with mean 1/γ. The symptomatic and asymptomatic individuals then move to the recovered classes ($R_{1}(t)$ or $R_{2}(t)$ respectively). Then, individuals in the recovered class $R_{1}(t)$ can progress to the vaccinated class $V_{1R}(t)$ if they get vaccinated. However, they can also progress (with different probabilities) to infected subpopulations $I_{1}(t),I_{2}(t),A_{1}(t)$ or $A_{2}(t)$, depending on which strain they get and the symptoms. Analogously, individuals in the recovered class $R_{2}(t)$ can progress to the vaccinated class $V_{2R}(t)$ if they get vaccinated and to the infected subpopulations $I_{1}(t),I_{2}(t),A_{1}(t)$ or $A_{2}(t)$. Recovered individuals cannot go back to the susceptible population due to partial cross immunity and adaptive immune system that has memory [57,124–128]. Finally, symptomatic individuals can die due to COVID-19, but the model assumes that people who are asymptomatic cannot. The model, as any other epidemiological model, is obviously a simplification of reality. For instance, the hospitalization subpopulation is not considered explicitly, nor are presymptomatic individuals. It is important to remark that a large number of studies assume these simplifications in order to focus on some particular stages and/or parameters.

The model allows us to analyze the dynamics of the Omicron wave taking into account two SARS-CoV-2 strains. Studies have shown that exposure to small airborne particles is equally, or even more, likely to lead to infection with SARS-CoV-2 as the more widely recognized transmission via larger respiratory droplets and/or direct contact with infected people or contaminated surfaces [129]. Thus, we can model the transmission of SARS-CoV-2 by mass action, i.e., a term βSI, where β is the SARS-CoV-2 transmission rate [117]. The total population size is given by

(1) $$N\left(t\right)=S\left(t\right)+I_{1}\left(t\right)+A_{1}\left(t\right)+I_{2}\left(t\right)+A_{2}\left(t\right)+R_{1}\left(t\right)+R_{2}\left(t\right)+V\left(t\right)+V_{1R}\left(t\right)+V_{2R}\left(t\right).$$

The total population N(t) does not include the cumulative deaths but we can compute them in the in silico simulations. The model is represented by the next differential equations

(2) $$\dot{S}(t)\;=\Lambda -\left(v+d\right)S\left(t\right)-\lambda_{1}\left(t\right)S\left(t\right)-\lambda_{2}\left(t\right)S\left(t\right),\;{\dot{I}}_{1}(t)\;=\left(1-a_{1}\right)\lambda_{1}(t)\left(S(t)+\left(1-\epsilon_{1}\right)V(t)+\left(1-\epsilon_{1R}\right)V_{1R}(t)+\left(1-\epsilon_{21R}\right)V_{2R}(t)\right.\;\left.+\left(1-\epsilon_{1}\right)R_{1}(t)+\left(1-\epsilon_{21}\right)R_{2}(t)\right)-\left(d+d_{1}+\gamma \right)I_{1}\left(t\right),\;\dot{A_{1}}(t)\;=a_{1}\lambda_{1}(t)\left(S(t)+\left(1-\epsilon_{1}\right)V(t)+\left(1-\epsilon_{1R}\right)V_{1R}(t)+\left(1-\epsilon_{21R}\right)V_{2R}(t)\right.\;\left.+\left(1-\epsilon_{1}\right)R_{1}(t)+\left(1-\epsilon_{21}\right)R_{2}(t)\right)-\left(d+\gamma \right)A_{1}\left(t\right),\;{\dot{I}}_{2}(t)\;=\left(1-a_{2}\right)\lambda_{2}(t)\left(S(t)+\left(1-\epsilon_{2}\right)V(t)+\left(1-\epsilon_{2R}\right)V_{2R}(t)+\left(1-\epsilon_{12R}\right)V_{1R}(t)\right.\;\left.+\left(1-\epsilon_{2}\right)R_{1}(t)+\left(1-\epsilon_{22}\right)R_{2}(t)\right)-\left(d+d_{2}+\gamma \right)I_{2}\left(t\right),\;{\dot{A}}_{2}(t)\;=a_{2}\lambda_{2}(t)\left(S(t)+\left(1-\epsilon_{2}\right)V(t)+\left(1-\epsilon_{2R}\right)V_{2R}(t)+\left(1-\epsilon_{12R}\right)V_{1R}(t)\right.\;\left.+\left(1-\epsilon_{2}\right)R_{1}(t)+\left(1-\epsilon_{22}\right)R_{2}(t)\right)-\left(d+\gamma \right)A_{2}\left(t\right),\;\dot{R_{1}}(t)\;=\gamma \left(I_{1}(t)+A_{1}(t)\right)-\left(d+v_{r}\right)R_{1}\left(t\right)-\lambda_{1}\left(t\right)\left(1-\epsilon_{1}\right)R_{1}\left(t\right)-\lambda_{2}\left(t\right)\left(1-\epsilon_{2}\right)R_{1}\left(t\right),\;\dot{R_{2}}(t)\;=\gamma \left(I_{2}(t)+A_{2}(t)\right)-\left(d+v_{r}\right)R_{2}(t)-\lambda_{2}(t)\left(1-\epsilon_{22}\right)R_{2}(t)-\lambda_{1}(t)\left(1-\epsilon_{21}\right)R_{2}(t),\;\dot{V}(t)\;=vS\left(t\right)-dV\left(t\right)-\left(1-\epsilon_{1}\right)\lambda_{1}\left(t\right)V\left(t\right)-\left(1-\epsilon_{2}\right)\lambda_{2}\left(t\right)V\left(t\right),\;\dot{V_{1R}}(t)\;=v_{r}R_{1}\left(t\right)-dV_{1R}\left(t\right)-\left(1-\epsilon_{1R}\right)\lambda_{1}\left(t\right)V_{1R}\left(t\right)-\left(1-\epsilon_{12R}\right)\lambda_{2}\left(t\right)V_{1R}\left(t\right),\;\dot{V_{2R}}(t)\;=v_{r}R_{2}\left(t\right)-dV_{2R}\left(t\right)-\left(1-\epsilon_{2R}\right)\lambda_{2}\left(t\right)V_{2R}\left(t\right)-\left(1-\epsilon_{21R}\right)\lambda_{1}\left(t\right)V_{2R}\left(t\right),$$

where $\lambda_{1}(t)=\beta_{I_{1}}I_{1}(t)+\beta_{A_{1}}A_{1}(t)$ and $\lambda_{2}(t)=\beta_{I_{2}}I_{2}(t)+\beta_{A_{2}}A_{2}(t)$ are the sources that produce infections in the different at risk subpopulations. The model comprises ten dependent variables, representing the different subpopulations. The parameters with their respective meaning and numerical values are shown in Table 1.

We will analyze some basic features of the model (2) in order to obtain a mathematical framework for the stability analysis. Some conditions of the model (2) are The initial conditions satisfy

(3) $$S(0)>0,I_{1}(0)\geq 0,A_{1}(0)\geq 0,I_{2}(0)\geq 0,A_{2}(0)\geq 0,R_{1}(0)\geq 0,R_{2}(0)\geq 0,\;V(0)>0,V_{1R}(0)\geq 0,V_{2R}(0)\geq 0.$$

The parameters satisfy

(4) $$\Lambda ,\beta_{{!}_{2}},\beta_{I_{1}},\beta_{A_{2}},\beta_{A_{1}},\alpha ,\gamma ,d,d_{i},v_{r}\in R^{+},\text{and}\;a_{i},\epsilon_{i},\epsilon_{iR},\epsilon_{ij},\epsilon_{ijR}\in \left[0,1\right].$$

### Positivity

By the classical theory of ordinary differential equations [135,136], it deduces that the system (2) is well-posed, and has a unique solution

$$𝒵(t)≔\left(S(t),I_{1}(t),A_{1}(t),I_{2}(t),A_{2}(t),R_{1}(t),R_{2}(t),V(t),V_{1R}(t),V_{2R}(t)\right)$$

satisfying the initial conditions given by (3). The dependent variables of the system (2) are subpopulations; therefore, we must show that if (3) holds, then the solutions of the mathematical model (2) are positive ∀t>0.

**Theorem 1.** *Assume that (2) and (3) hold. Then the solution* 𝒵(t) *of (2) is positive and uniformly bounded* ∀t>0.

**Proof.** We define the following number

$$𝒲=sup\left\{\rho >0/\forall t\in [0,\rho ],S(t)>0,I_{i}(t)\geq 0,A_{i}(t)\geq 0,R_{i}(t)\geq 0,V(t)>0,V_{1R}(t)\geq 0,V_{2R}(t)\geq 0\right\},$$

for i=1,2. Suppose that 𝒲<∞. Since the solutions of the model (2) are continuous, it follows that

$$S(𝒲)=0,\;\text{or}\;I_{2}(𝒲)=0,\;\text{or}\;I_{1}(𝒲)=0,\;\text{or}\;A_{2}(𝒲)=0,\;\text{or}\;A_{1}(𝒲)=0,\;\text{or}\;R_{1}(𝒲)=0,\;\text{or}\;R_{2}(𝒲)=0,\;\text{or}\;V(𝒲)=0,\;\text{or}\;V_{1R}(𝒲)=0,\;\text{or}\;V_{2R}(𝒲)=0.$$

Thus, if S(𝒲)=0, is obtained before the other variables, one obtains

$$\frac{dS(𝒲)}{dt}=\underset{t\to {𝒲}^{-}}{lim}\;\frac{S(𝒲)-S(t)}{𝒲-t}\leq 0.$$

Accordingly, from first the Equation of the model (2), one obtains that

$$\dot{S}(𝒲)\;=\Lambda -(v+d)S(𝒲)-\lambda_{1}(𝒲)S(𝒲)+\lambda_{2}(𝒲)S(𝒲)\;=\Lambda >0,$$

which is a contradiction. Therefore, S(t)>0, for all t≥0. Now, similarly, if we assume that V(𝒲)=0, occurs before any of the other variables are zero, one obtains

$$\frac{dV(𝒲)}{dt}=\underset{t\to {𝒲}^{-}}{lim}\;\frac{V(𝒲)-V(t)}{𝒲-t}\leq 0,$$

and using the eighth Equation (2), another contradiction follows

$$\dot{V}(𝒲)=vS(𝒲)-dV(𝒲)-\left(1-\epsilon_{1}\right)\lambda_{1}(t)V(𝒲)-\left(1-\epsilon_{2}\right)\lambda_{2}(t)V(𝒲)>0.$$

We can use a similar process for the other dependent variables to obtain to similar contradictions. Therefore, 𝒲=+∞, and therefore

$$S(t)\geq 0,I_{1}(t)\geq 0,A_{1}(t)\geq 0,I_{2}(t)\geq 0,A_{2}(t)\geq 0,R_{1}(t)\geq 0,R_{2}(t)\geq 0,V(t)\geq 0,\;V_{1R}(t)\geq 0,V_{2R}(t)\geq 0,$$

for t>0. Next, using (2) one obtains

(5) $$\dot{N}\left(t\right)=\Lambda -dN\left(t\right)-d_{1}I_{1}\left(t\right)-d_{2}I_{2}\left(t\right)\leq \Lambda -dN\left(t\right),$$

and using Gronwall inequalities one obtains that

(6) $$N\left(t\right)\leq \frac{\Lambda }{d}+\left(N\left(0\right)-\frac{\Lambda }{d}\right)e^{-dt},$$

for t≥0. Now, taking $N(0)\leq \frac{\Lambda }{d}$, then $N(t)\leq \frac{\Lambda }{d}$. On the other hand, from the first and eighth Eqs. of system (2) it follows that

$$\dot{S}\left(t\right)=\Lambda -\left(v+d\right)S\left(t\right)-\lambda_{1}\left(t\right)S\left(t\right)-\lambda_{2}\left(t\right)S\left(t\right)\leq \Lambda -\left(v+d\right)S\left(t\right),$$

and

$$\dot{V}\left(t\right)=vS\left(t\right)-dV\left(t\right)-\left(1-\epsilon_{1}\right)\lambda_{1}\left(t\right)V\left(t\right)-\left(1-\epsilon_{2}\right)\lambda_{2}\left(t\right)V\left(t\right)\leq \nu S\left(t\right)-dV\left(t\right).$$

Taking the limit, we have that $S(t)\leq \frac{\Lambda }{v+d}$ and $V(t)\leq \frac{v\Lambda }{d(v+d)}$ as t→∞. As a result, θ∈[0,1) implies that

$$0<S\left(t\right)+\theta V\left(t\right)\leq \frac{\Lambda \left[d+\theta v\right]}{d\left(d+v\right)},\;\text{as}\;t\to \infty .$$

Therefore, we can consider the region

(7) $$𝒪=\left\{\begin{array}{c} \left(S,I_{1},A_{1},I_{2},A_{2},R_{1},R_{2},V,V_{1R},V_{2R}\right)\in R_{+}^{10}\left|\begin{array}{c} N(t)\leq \frac{\Lambda }{d},S(t)\leq \frac{\Lambda }{d(v+d)},\\ 0<S(t)+\theta V(t)\leq \frac{\Lambda [d+\theta v]}{d(d+v)},\theta \in [0,1) \end{array}\right. \end{array}\right\}$$

which is positively invariant. Thus, the solutions of system (2) are bounded. Furthermore, if $N(0)>\frac{\Lambda }{d}$, then either the solution enters 𝒪 for infinite time or $N(t)\to \frac{\Lambda }{d}$ asymptotically. **☐**

## 3. Stability Analysis

In the qualitative analysis of the model solutions, it is common to determine the stationary points that identify the disease-free and endemic equilibrium points. In this case, in the model (2) there is a disease-free point $\left(F_{1}^{*}\right)$, which can be found by setting $I_{1}=I_{2}=A_{1}=A_{2}=0$, and indicates that SARS-CoV-2 becomes extinct. Now, it is of great importance to determine in epidemiological models the different parameters that delimit the different states of a disease. One in particular is the basic reproduction number ${ℛ}_{0}$, which measures the influence of introducing one infected individual into a total susceptible population [76,137].

### 3.1. Disease-Free Equilibrium Point and ${ℛ}_{0}$

The disease-free equilibrium $\left(F_{1}^{*}\right)$ point of the model (2) is given by

(8) $$F_{1}^{*}=\left(S^{0},I_{1}^{0},A_{1}^{0},I_{2}^{0},A_{2}^{0},R_{1}^{0},R_{2}^{0},V^{0},V_{1R}^{0},V_{2R}^{0}\right)=\left(\frac{\Lambda }{d+v},0,0,0,0,0,0,0,\frac{v\Lambda }{d(d+v)},0,0\right).$$

In order to obtain an expression for ${ℛ}_{0}$ in the model (2), we use the next generation matrix (NGM) method [116,137]. For this purpose, we determine the matrix ℱ representing the new infection cases and the matrix 𝒱 represents the progression between classes. The eigenvalue of the matrix $ℱ{𝒱}^{-1}$ with largest absolute value is the basic reproduction number ${ℛ}_{0}$. For further technicalities see [116,137]. Thus,

(9) $$ℱ=\left[\begin{array}{cccc} \left(1-a_{1}\right)B_{I_{1}} & \left(1-a_{1}\right)B_{A_{1}} & 0 & 0\\ a_{1}B_{I_{1}} & a_{1}B_{A_{1}} & 0 & 0\\ 0 & 0 & \left(1-a_{2}\right)B_{I_{2}} & \left(1-a_{2}\right)B_{A_{2}}\\ 0 & 0 & a_{2}B_{I_{2}} & a_{2}B_{A_{2}} \end{array}\right],$$

and

(10) $$𝒱=\left[\begin{array}{cccc} d+d_{1}+\gamma & 0 & 0 & 0\\ 0 & d+\gamma & 0 & 0\\ 0 & 0 & d+d_{2}+\gamma & 0\\ 0 & 0 & 0 & d+\gamma \end{array}\right].$$

Then, one obtains

$$ℱ{𝒱}^{-1}=\left[\begin{array}{cccc} \frac{\left(1-a_{1}\right)B_{I_{1}}}{d+d_{1}+\gamma } & \frac{\left(1-a_{1}\right)B_{A_{1}}}{d+\gamma } & 0 & 0\\ \frac{a_{1}B_{I_{1}}}{d+d_{1}+\gamma } & \frac{a_{1}B_{A_{1}}}{d+\gamma } & 0 & 0\\ 0 & 0 & \frac{\left(1-a_{2}\right)B_{I_{2}}}{d+d_{2}+\gamma } & \frac{\left(1-a_{2}\right)B_{A_{2}}}{d+\gamma }\\ 0 & 0 & \frac{a_{2}B_{I_{2}}}{d+d_{2}+\gamma } & \frac{a_{2}B_{A_{2}}}{d+\gamma } \end{array}\right],$$

which is the NGM, and the positive eigenvalues are given by

(11) $${ℛ}_{0_{1}}=\frac{B_{A_{1}}a_{1}\left(d+d_{1}+\gamma \right)+B_{I_{1}}\left(1-a_{1}\right)(d+\gamma )}{(d+\gamma )\left(d+d_{1}+\gamma \right)},\;{ℛ}_{0_{2}}=\frac{B_{A_{2}}a_{2}\left(d+d_{2}+\gamma \right)+B_{I_{2}}\left(1-a_{2}\right)(d+\gamma )}{(d+\gamma )\left(d+d_{2}+\gamma \right)},$$

or

$${ℛ}_{0_{1}}=\frac{B_{A_{1}}a_{1}}{d+\gamma }+\frac{B_{I_{1}}\left(1-a_{1}\right)}{d+d_{1}+\gamma },\;{ℛ}_{0_{2}}=\frac{B_{A_{2}}a_{2}}{d+\gamma }+\frac{B_{I_{2}}\left(1-a_{2}\right)}{d+d_{2}+\gamma },$$

where

$$B_{I_{1}}=\frac{\beta_{I_{1}}\Lambda }{v+d}\left(1+\frac{\left(1-\epsilon_{1}\right)v}{d}\right),B_{A_{1}}=\frac{\beta_{A_{1}}\Lambda }{v+d}\left(1+\frac{\left(1-\epsilon_{1}\right)v}{d}\right),\;B_{I_{2}}=\frac{\beta_{I_{2}}\Lambda }{v+d}\left(1+\frac{\left(1-\epsilon_{2}\right)v}{d}\right),B_{A_{2}}=\frac{\beta_{A_{2}}\Lambda }{v+d}\left(1+\frac{\left(1-\epsilon_{2}\right)v}{d}\right).$$

The parameters ${ℛ}_{0_{1}}$ and ${ℛ}_{0_{2}}$ are related to the two different SARS-CoV-2 strains, respectively. Thus, one obtains the spectral radius of $ℱV^{-1}$

(12) $${ℛ}_{0}=max\left\{{ℛ}_{0_{1}},{ℛ}_{0_{2}}\right\}.$$

The parameter ${ℛ}_{0}$ allows us to determine if an outbreak would occur. When ${ℛ}_{0}<1$, and if the initial conditions of the model (2) are close enough to the equilibrium $\left(F_{1}^{*}\right)$, then no outbreak would occur. However, when ${ℛ}_{0}>1$, an epidemic would occur. Thus, one obtains the next theorem.

**Theorem 2.** *When the basic reproduction number ${ℛ}_{0}<1\left({ℛ}_{0}>1\right)$*, *the disease-free equilibrium point* $F_{1}^{*}$ *of the model (2) and given in (8) is locally asymptotically stable (unstable)*.

**Proof.** The proof follows from applying Theorem 2 in [137]. ☐

Global Stability of Disease-Free Equilibrium Point

Analyzing the behavior of the solutions of an epidemiological model represented by a system of differential equations such as (2) around the disease-free equilibrium point is important because it determines what public health measures are necessary in order to avoid endemic situations. Thus, we want to analyze whether the disease-free point $F_{1}^{*}$ is a global attractor, i.e., it must be proven that if ${ℛ}_{0}<1$, the disease becomes extinct regardless of the initial conditions of the model (2). In other words, the point $F_{1}^{*}$ is globally asymptotically stable (GAS). In order to prove the global stability of $F_{1}^{*}$, we apply the methodology used in [138]. The system (2) can be written as

(13) $$\dot{Y}\left(t\right)=F\left(Y,Z\right),\;\dot{Z}\left(t\right)=I\left(Y,Z\right),\;I\left(Y,0\right)=0\in R^{4},$$

with $Y=\left(S,V,R_{1},R_{2},V_{R1},V_{R2}\right)$ which denotes the vector of uninfected compartments, and $Z=\left(I_{1},A_{1},I_{2},A_{2}\right)$ is the vector of infected compartments. Moreover, F(Y,0) is the right-hand side of $\dot{S}(t),\dot{V}(t),{\dot{R}}_{1}(t),{\dot{R}}_{2}(t),{\dot{V}}_{R1}(t),{\dot{V}}_{R2}(t)$, setting $I_{1}=A_{1}=I_{2}=A_{2}=0$. Thus, $F_{1}^{*}$ is rewritten as $Y^{0}=\left(S^{0},V^{0},0\right),0\in R^{4}$. The following result guarantees the GAS of $F_{1}^{*}$.

**Theorem 3.** *The point* $F_{1}^{*}$ *given by (8) of system (2) is GAS in* 𝒪 *if* ${ℛ}_{0}\leq 1$, a*nd if the next conditions hold:*

- **Condition 1** : *Given* $\dot{Y}(t)=F(Y,0),0\in R^{4}$, *then* $Y^{0}$ *is GAS*.
- **Condition 2** : $I(Y,Z)=𝒥Z-\overset{\circ }{I}(Y,Z)$, *then* $\overset{\circ }{I}(Y,Z)\geq 0$ *in* 𝒪 *as* t→∞, *and* $𝒥=D_{Z}\left(\overset{\circ }{I},0\right)$ *is an* M*–matrix, i.e., the off-diagonal elements are non-negative*.

**Proof.** For the **Condition 1**, we write $\dot{Y}(t)=F(Y,0),0\in R^{4}$ as

(14) $$\dot{S}(t)\;=\Lambda -\left(v+d\right)S\left(t\right),\;\dot{V}(t)\;=vS\left(t\right)-dV\left(t\right),\;\dot{R_{1}}(t)\;=-\left(d+v_{r}\right)R_{1}\left(t\right),\;\dot{R_{2}}(t)\;=-\left(d+v_{r}\right)R_{2}\left(t\right),\;\dot{V_{1R}}(t)\;=v_{r}R_{1}\left(t\right)-dV_{1R}\left(t\right),\;\dot{V_{2R}}(t)\;=v_{r}R_{2}\left(t\right)-dV_{2R}\left(t\right).$$

After some calculations using (14) one obtains

(15) $$\left(S(t),V(t),R_{1}(t),R_{2}(t),dV_{1R}(t),V_{2R}(t)\right)\to \left(S^{0},V^{0},0\right)\;\text{as}\;t\to \infty .$$

On the other hand, for the **Condition 2**, from (9) and (10) we can obtain the matrix 𝒥=ℱ-𝒱.

$$𝒥=\left[\begin{array}{cccc} \left(1-a_{1}\right)B_{I_{1}}-\left(d+d_{1}+\gamma \right) & \left(1-a_{1}\right)B_{A_{1}} & 0 & 0\\ a_{1}B_{I_{1}} & a_{1}B_{A_{1}}-(d+\gamma ) & 0 & 0\\ 0 & 0 & \left(1-a_{2}\right)B_{I_{2}}-\left(d+d_{2}+\gamma \right) & \left(1-a_{2}\right)B_{A_{2}}\\ 0 & 0 & a_{2}B_{I_{2}} & a_{2}B_{A_{2}}-(d+\gamma ) \end{array}\right],$$

and 𝒥 is an M–matrix. Next, from (15) and in view of (7) yields

$$\overset{\circ }{I}(Y,Z)=𝒥Z-I(Y,Z)=\left[\begin{array}{c} \left\{\frac{\Lambda \left(d+\left(1-\epsilon_{1}\right)v\right)}{d\left(d+v\right)}-\left(S+\left(1-\epsilon_{1}\right)V\right)\right\}\left(1-a_{1}\right)\lambda_{1}-\left(1-a_{1}\right)\lambda_{1}W_{1}\\ \left\{\frac{\Lambda \left(d+\left(1-\epsilon_{1}\right)v\right)}{d\left(d+v\right)}-\left(S+\left(1-\epsilon_{1}\right)V\right)\right\}a_{1}\lambda_{1}-a_{1}\lambda_{1}W_{1}\\ \left\{\frac{\Lambda \left(d+\left(1-\epsilon_{2}\right)v\right)}{d\left(d+v\right)}-\left(S+\left(1-\epsilon_{2}\right)V\right)\right\}\left(1-a_{12}\right)\lambda_{2}-\left(1-a_{2}\right)\lambda_{2}W_{2}\\ \left\{\frac{\Lambda \left(d+\left(1-\epsilon_{2}\right)v\right)}{d\left(d+v\right)}-\left(S+\left(1-\epsilon_{2}\right)V\right)\right\}a_{2}\lambda_{2}-a_{2}\lambda_{2}W_{2} \end{array}\right]\geq 0,$$

in Ω as t→∞, where

$$W_{1}\left(t\right)=\left[\left(1-\epsilon_{1R}\right)V_{1R}\left(t\right)+\left(1-\epsilon_{21R}\right)V_{2R}\left(t\right)+\left(1-\epsilon_{1}\right)R_{1}\left(t\right)+\left(1-\epsilon_{21}\right)R_{2}\left(t\right)\right],$$

and

$$W_{2}\left(t\right)=\left(1-\epsilon_{2R}\right)V_{2R}\left(t\right)+\left(1-\epsilon_{12R}\right)V_{1R}\left(t\right)+\left(1-\epsilon_{2}\right)R_{1}\left(t\right)+\left(1-\epsilon_{22}\right)R_{2}\left(t\right),$$

with $W_{1}(t),W_{2}(t)\to 0$, as t→∞. Thus, it is very clear that $\overset{\circ }{I}(Y,Z)\geq 0$, with $0\in R^{4}$. **☐**

The consequence of Theorem 3 from the epidemiological viewpoint is that COVID will not become endemic as long as ${ℛ}_{0}<1$, regardless of the initial conditions.

### 3.2. Endemic Equilibrium Point

The behavior of the solutions of the model (2) when ${ℛ}_{0}>1$ depends on the endemic points. We can find these endemic points by simply setting the right-hand side of the system (2) to zero and obtaining the algebraic solutions representing the endemic points as a function of the parameters of the mathematical model (2).

For the model (2), we want to determine the endemic points, which will be denoted by

(16) $$E^{*}=\left(S^{*},A_{1}^{*},I_{1}^{*},A_{2}^{*},I_{2}^{*},R_{1}^{*},R_{2}^{*},V^{*},V_{1R}^{*},V_{2R}^{*}\right),$$

and this vector is a solution of the following algebraic system:

(17) $$0=\Lambda -\left(v+d\right)S^{*}-\lambda_{1}^{*}S^{*}-\lambda_{2}^{*}S^{*},\;0=\left(1-a_{1}\right)\lambda_{1}^{*}\left(S^{*}+\left(1-\epsilon_{1}\right)V^{*}+\left(1-\epsilon_{1R}\right)V_{1R}^{*}+\left(1-\epsilon_{21R}\right)V_{2R}^{*}+\left(1-\epsilon_{1}\right)R_{1}^{*}\right.\;\left.+\left(1-\epsilon_{21}\right)R_{2}^{*}\right)-\left(d+d_{1}+\gamma \right)I_{1}^{*},\;0=a_{1}\lambda_{1}^{*}\left(S^{*}+\left(1-\epsilon_{1}\right)V^{*}+\left(1-\epsilon_{1R}\right)V_{1R}^{*}+\left(1-\epsilon_{21R}\right)V_{2R}^{*}+\left(1-\epsilon_{1}\right)R_{1}^{*}\right.\;\left.+\left(1-\epsilon_{21}\right)R_{2}^{*}\right)-\left(d+\gamma \right)A_{1}^{*},\;0=\left(1-a_{2}\right)\lambda_{2}^{*}\left(S^{*}+\left(1-\epsilon_{2}\right)V^{*}+\left(1-\epsilon_{2R}\right)V_{2R}^{*}+\left(1-\epsilon_{12R}\right)V_{1R}^{*}+\left(1-\epsilon_{2}\right)R_{1}^{*}\right.\;\left.+\left(1-\epsilon_{22}\right)R_{2}^{*}\right)-\left(d+d_{2}+\gamma \right)I_{2}^{*},\;0=a_{2}\lambda_{2}^{*}\left(S^{*}+\left(1-\epsilon_{2}\right)V^{*}+\left(1-\epsilon_{2R}\right)V_{2R}^{*}+\left(1-\epsilon_{12R}\right)V_{1R}^{*}+\left(1-\epsilon_{2}\right)R_{1}^{*}\right.\;\left.+\left(1-\epsilon_{22}\right)R_{2}^{*}\right)-(d+\gamma )A_{2}^{*},\;0=\gamma \left(I_{1}^{*}+A_{1}^{*}\right)-\left(d+v_{r}\right)R_{1}^{*}-\lambda_{1}^{*}\left(1-\epsilon_{1}\right)R_{1}^{*}-\lambda_{2}^{*}\left(1-\epsilon_{2}\right)R_{1}^{*},\;0=\gamma \left(I_{2}^{*}+A_{2}^{*}\right)-\left(d+v_{r}\right)R_{2}^{*}-\lambda_{2}^{*}\left(1-\epsilon_{22}\right)R_{2}^{*}-\lambda_{1}^{*}\left(1-\epsilon_{21}\right)R_{2}^{*},\;0=vS^{*}-dV^{*}-\left(1-\epsilon_{1}\right)\lambda_{1}^{*}V^{*}-\left(1-\epsilon_{2}\right)\lambda_{2}^{*}V^{*},\;0=v_{r}R_{1}^{*}-dV_{1R}^{*}-\left(1-\epsilon_{1R}\right)\lambda_{1}^{*}V_{1R}^{*}-\left(1-\epsilon_{12R}\right)\lambda_{2}^{*}V_{1R}^{*},\;0=v_{r}R_{2}^{*}-dV_{2R}^{*}-\left(1-\epsilon_{2R}\right)\lambda_{2}^{*}V_{2R}^{*}-\left(1-\epsilon_{21R}\right)\lambda_{1}^{*}V_{2R}^{*},$$

where $\lambda_{1}^{*}=\beta_{I_{1}}I_{1}^{*}+\beta_{A_{1}}A_{1}^{*}$ and $\lambda_{2}^{*}=\beta_{I_{2}}I_{2}^{*}+\beta_{A_{2}}A_{2}^{*}$. We can see from the first Eq. of the system (17) that $S^{*}>0$. Moreover, $\Lambda -(d+v)S^{*}>0$, that is, $S^{*}\in 𝒪$. Using the second, third, fourth and fifth Equation (17) we arrive to the next result,

(18) $$I_{1}^{*}=\frac{\left(1-a_{1}\right)(d+\gamma )A_{1}^{*}}{a_{1}\left(d+d_{1}+\gamma \right)},I_{2}^{*}=\frac{\left(1-a_{2}\right)(d+\gamma )A_{2}^{*}}{a_{2}\left(d+d_{2}+\gamma \right)}.$$

Thus

(19) $$\lambda_{1}^{*}=\frac{d{ℛ}_{0_{1}}(d+\gamma )(v+d)A_{1}^{*}}{a_{1}\Lambda \left(d+\left(1-\epsilon_{1}\right)v\right)},\lambda_{2}^{*}=\frac{d{ℛ}_{0_{2}}(d+\gamma )(v+d)A_{2}^{*}}{a_{2}\Lambda \left(d+\left(1-\epsilon_{2}\right)v\right)}.$$

Now, from the first Equation (17) it follows that

(20) $$S^{*}=\frac{\Lambda }{(v+d)\left(1+\lambda_{1}^{*}+\lambda_{2}^{*}\right)}.$$

Next, from the sixth and seventh Equation (17), and putting (18), it follows that

(21) $$R_{1}^{*}=\frac{\gamma \left(1+\frac{\left(1-a_{1}\right)(d+\gamma )}{a_{1}\left(d+d_{1}+\gamma \right)}\right)A_{1}^{*}}{\left(d+v_{r}+\lambda_{1}^{*}\left(1-\epsilon_{1}\right)+\lambda_{2}^{*}\left(1-\epsilon_{2}\right)\right)},R_{2}^{*}=\frac{\gamma \left(1+\frac{\left(1-a_{2}\right)(d+\gamma )}{a_{2}\left(d+d_{2}+\gamma \right)}\right)A_{2}^{*}}{\left(d+v_{r}+\lambda_{1}^{*}\left(1-\epsilon_{22}\right)+\lambda_{2}^{*}\left(1-\epsilon_{21}\right)\right)}$$

In the same way, from the ninth and tenth Equation (17), one obtains

(22) $$V_{1R}^{*}=\frac{v_{r}R_{1}^{*}}{\left(d+\lambda_{1}^{*}\left(1-\epsilon_{1R}\right)+\lambda_{2}^{*}\left(1-\epsilon_{12R}\right)\right)},V_{2R}^{*}=\frac{\nu_{r}R_{2}^{*}}{\left(d+\lambda_{1}^{*}\left(1-\epsilon_{2R}\right)+\lambda_{2}^{*}\left(1-\epsilon_{21R}\right)\right)},$$

and finally

(23) $$V^{*}=\frac{S^{*}v}{d+\left(1-\epsilon_{1}\right)\lambda_{1}^{*}+\left(1-\epsilon_{2}\right)\lambda_{2}^{*}}.$$

Thus, there are three endemic equilibrium points that can be obtained from Equation (18). Indeed, if $A_{1}^{*}=0$ and $A_{2}^{*}>0$, then one obtains from Equations (18)–(23) that $\lambda_{1}^{*}=0,\lambda_{2}^{*}>0,S^{*}>0,R_{1}^{*}=0,R_{2}^{*}>0,V_{1R}^{*}=0,V_{2R}^{*}>0$, and $V^{*}>0$. Thus, the first endemic point given by

(24) $$E_{1}^{*}=\left(S^{*},0,0,A_{2}^{*},I_{2}^{*},0,R_{2}^{*},V^{*},0,V_{2R}^{*}\right),.$$

Next, if $A_{2}^{*}=0$ and $A_{1}^{*}>0$, then one obtains from Equations (18)–(23) that $\lambda_{2}^{*}=0,\;\lambda_{1}^{*}>0,S^{*}>0,R_{2}^{*}=0,R_{1}^{*}>0,V_{2R}^{*}=0,V_{1R}^{*}>0$, and $V^{*}>0$. Therefore, the second endemic point is

(25) $$E_{2}^{*}=\left(S^{*},A_{1}^{*},I_{1}^{*},0,0,R_{1}^{*},0,V^{*},V_{1R}^{*},0\right).$$

Finally, if $A_{1}^{*}>0$ and $A_{2}^{*}>0$ then we can obtain the third endemic point given by Equations (18)–(23).

Thus, the steady states are one of the endemic equilibrium points depending on the numerical values of ${ℛ}_{0_{2}}$ and ${ℛ}_{0_{1}}$. For instance, if ${ℛ}_{0_{2}}>{ℛ}_{0_{1}}>1$ then both SARS-CoV-2 strains survive in the population. This is due to the fact that the mathematical model (2) does not consider full immunity either from vaccination or natural immunity [62,80]. Recent studies suggest that this is true for the COVID-19 pandemic situation [22,139–143]. We did not perform further stability analysis related to periodic solutions, backward bifurcations and global stability since the aim of this study is the short dynamics of the Omicron wave and obtaining further insight into it.

## 4. Simulations for the Omicron Wave

We performed in silico simulations of the Omicron wave model (2) for a variety of scenarios (in fact, infinitely many) in order to obtain insight into the Omicron wave situation and additional potential consequences of the Omircon strain on the dynamics of this pandemic. We varied the vaccine’s efficacy against the Omicron strain in order to consider, as some articles have mentioned, that the efficacy of the vaccine is lower against the Omicron strain [22,48]. We also varied the transmissibility and severity of the Omicron strain since it has been revealed that both factors significantly differ in comparison to the previous circulating SARS-CoV-2 strains [22,24,48]. The in silico simulations allow us to explain, at least partially, the Omicron wave period. We focus here on the qualitative results of the in silico simulations since there are uncertainties that make it very difficult to have accurate forecasts as time has proven over the COVID-19 waves.

The dependence of the transmission rate on the natural daily variability in human behavior makes estimation of the transmission rate very difficult. Sensitivity analysis is one means researchers often use to approach the uncertainties in the COVID-19 pandemic. The numerical simulations presented in the present study show different potential situations in order to remark on the distinct possibilities regarding the transmission rates. For instance, when the Omicron variant arose, the scientific community did not know if it was more transmissible or deadly than the previous strain. The simulations also have the aim of corroborating the theoretical results in addition to potential explanations of what happened in the real world. The simulations allow us to present different scenarios regarding the real values of transmission rate and case fatality rate. This provides additional insight regarding the COVID-19 pandemic dynamics and future scenarios for new variants.

All numerical simulations were carried out in Python 3.8. Ordinary differential equations were solved using the scipy.odeint routine. The simulations were performed with a PC (Intel(R) Core(TM) i7-7820HQ CPU, 2.90 GHz) with 64 Mb RAM. Table 1 shows the numerical values of the parameters that were used for the in silico simulations. For some parameters, we used a wide range of values in order to consider a larger number of scenarios and potentially extreme cases that might arise due to uncertainty in the parameters. For the initial subpopulations, we took the values from the particular situation of the USA just before the start of the Omicron wave period [1]. Based on previous works, the Omicron wave started around mid-November [45]. The values of the initial conditions can be extracted from different data sources. Like the CDC, we considered the possibility that for every symptomatic infected case there would be one asymptomatic case, even though there is some uncertainty for this [1,4,133]. We chose the situation of the USA since the reported data are more reliable than in other countries and the population is large enough to observe the main effects on the Omicron wave dynamics. In the numerical simulated scenarios, there is an effective reproduction number ${ℛ}_{t}$ that decreases as the susceptible subpopulation decreases [80,144]. During the in silico simulations, we assumed that the parameters are time-invariant, despite that in reality some parameters might vary over time. Introducing time-varying parameters is a difficult task although some modelers have attempted it [101]. For the percentage of asymptomatic cases we considered 50%, which is a situation proposed by the CDC [133]. Making reasonable changes to this percentage does not affect the qualitative conclusions of this study.

### 4.1. Efficacy of the Vaccine against the Omicron Strain

The Omicron strain has been detected in many countries [145]. Preliminary data related to the efficacies of current vaccines against the Omicron strain are available. It has been revealed that these efficacies are different in comparison with other SARS-CoV-2 strains. In [145], the authors analyzed 133,437 PCR test results and found that during the proxy Omicron period the vaccine efficacy against hospitalization was 70%, which is much lower than the 93% efficacy for the comparator period. In [52], the authors carried out a narrative review from 32 scientific articles supporting the idea that Omicron evades antibodies induced by primary vaccination or by SARS-CoV-2 infection. We use this information in order to set the efficacies of the vaccines for the numerical simulations. Based on several scientific articles, we assume that the current vaccines have less efficacy against the Omicron strain [24,51,128,146–148]. On the other hand, it has been revealed that the Omicron pseudovirus infects cells more efficiently than other SARS-CoV-2 strains [128]. Furthermore, those who received two doses of vaccine have lower neutralizing activity against Omicron [22].

Table 2 shows the different efficacies of the vaccines for a variety of status related to COVID-19. Some of these efficacies are high if the individuals already had the disease in good agreement with previous studies [149,150]. Due to a short time study of less than one year, the model does not consider a particular subpopulation for the cases where individuals contracted the disease twice, which is very unlikely. However, the model can also be used as an approximation for longer times, since it considers that once individuals have been infected with SARS-CoV-2, the likelihood to get infected again is lower due to memory cells and adaptive immunity [151–153]. The model considers implicitly the waning of the effectiveness of the vaccine as well as natural immunity since vaccinated and recovered people can get infected but with lower probability [153–155].

### 4.2. Numerical Simulations towards Steady States

We present three in silico simulations of the model (2) in order to provide additional support to the theoretical results and observe the long-term behavior. For these scenarios we used initial conditions where the number of infected cases is very small since we just want to compare with the theoretical results and since ${ℛ}_{0}$ is defined for almost fully susceptible populations [116,137]. We varied the transmissibility of circulating SARS-CoV-2 strains and we considered that the Omicron strain has a higher likelihood to be transmitted than the previously circulating strain. This allows us to foresee the long-term qualitative effects of the Omicron wave.

**Figure 2.**
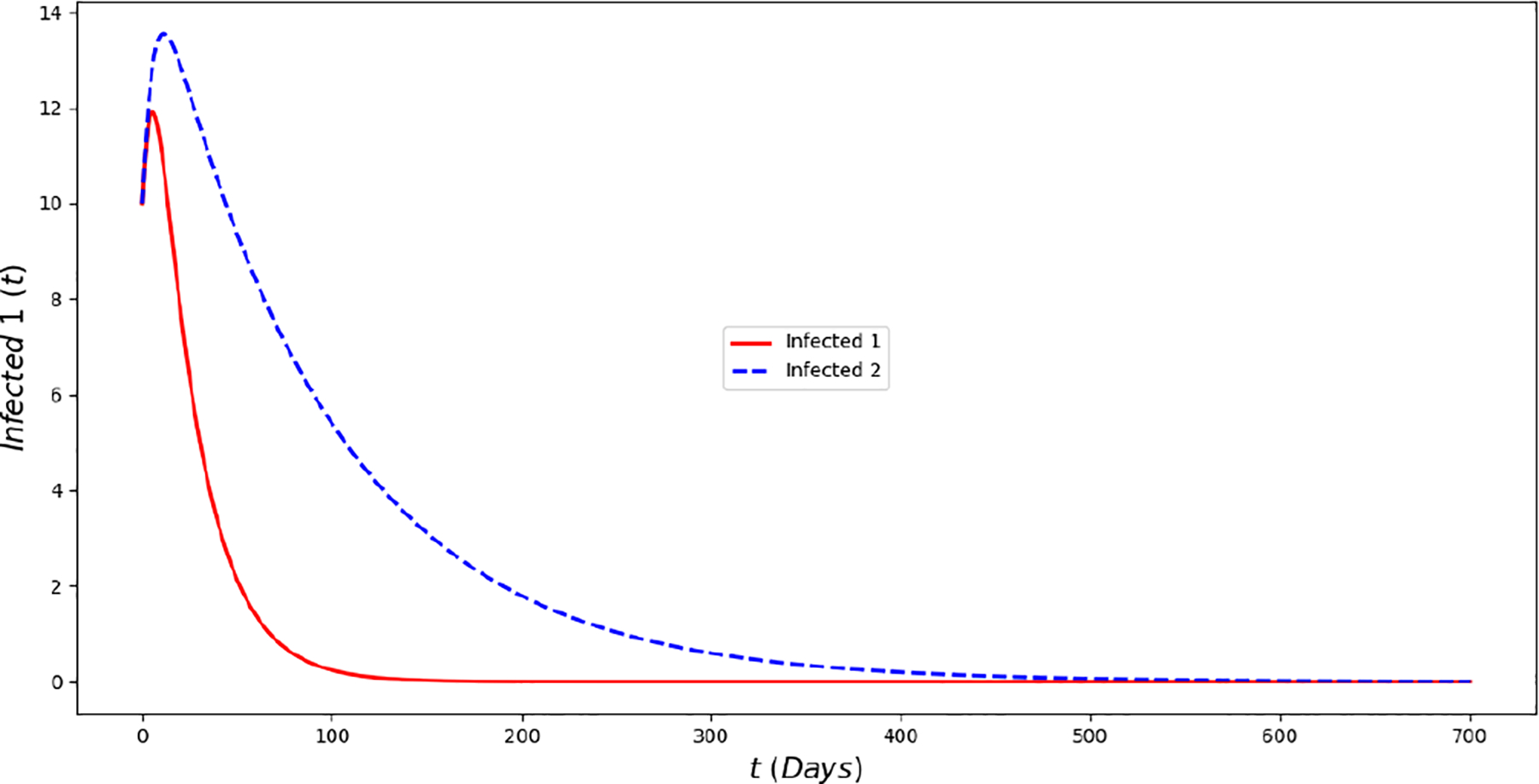
In silico simulation of the Omicron wave model (2) when ${ℛ}_{0_{2}}\approx 0.95>{ℛ}_{0_{1}}\approx 0.82$. The previously circulating and Omicron strains disappear, while the system approaches the point $F_{1}^{*}$. We use a large natural death rate for faster dynamics.

**Figure 3.**
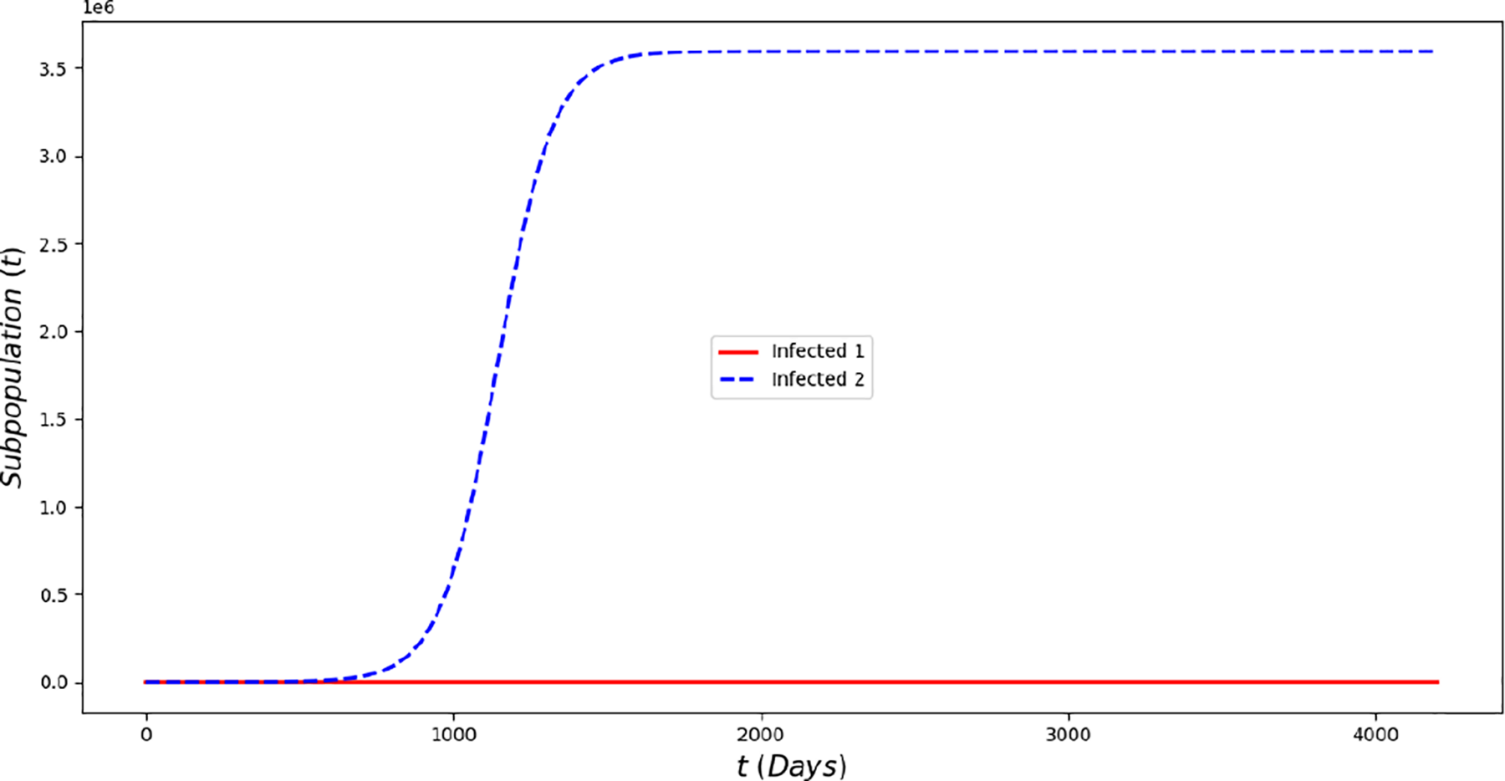
In silicosimulations of the Omicron wave model (2) when ${ℛ}_{0_{2}}\approx 1.04>{ℛ}_{0_{1}}\approx 0.9$. The Omicron strain becomes prevalent and the system approaches the point $E_{1}^{*}$.

**Figure 4.**
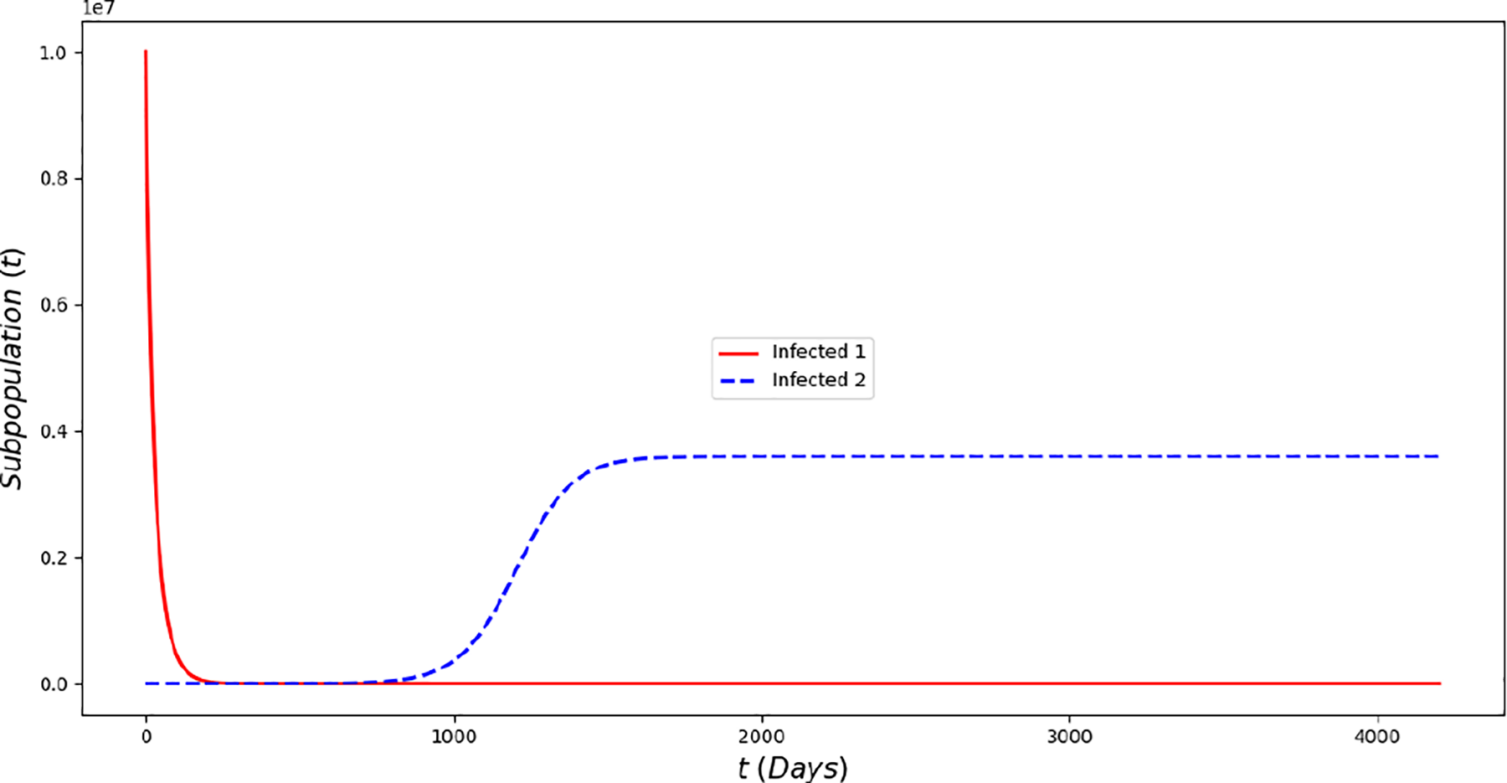
In silico of the Omicron wave model (2) when ${ℛ}_{0_{2}}\approx 1.04>{ℛ}_{0_{1}}\approx 0.9$. The Omicron strain becomes prevalent and the system approaches the endemic steady state $E_{1}^{*}$ despite the fact that the initial prevalence of the non-Omicron strain has a very large prevalence.

**Figure 5.**
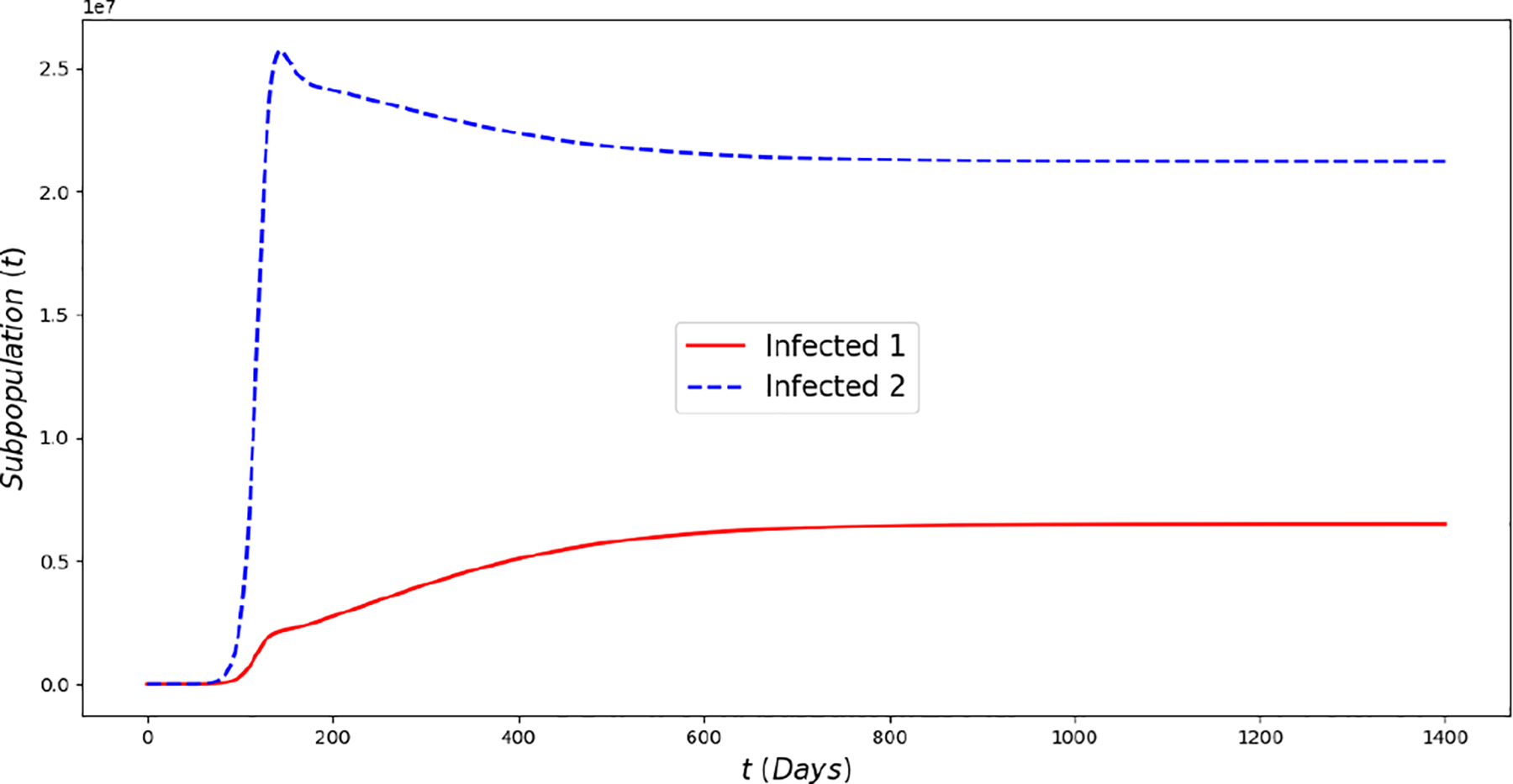
In silico simulations of the Omicron wave model (2) when ${ℛ}_{0_{2}}\approx 1.5>{ℛ}_{0_{1}}\approx 1.4$. The previously circulating and Omicron strains become prevalent and the system approaches the endemic steady state $E^{*}$.

Figure 2 displays the evolution of the symptomatic subpopulations $I_{1}(t)$ and $I_{2}(t)$. We chose the transmission rate such that ${ℛ}_{0_{1}}<1$ and ${ℛ}_{0_{2}}<1$. Both symptomatic (the asymptomatic cases were also treated but are not shown) subpopulations approach the disease-free steady state $F_{1}^{*}$. In order to obtain manageable and useful graphs for the steady states we use a large natural death rate for faster dynamics only in this subsection. Figure 3 displays the long-term behavior when ${ℛ}_{0_{2}}>1>{ℛ}_{0_{1}}$ and the initial infected subpopulations are small. Note that the Omicron strain becomes the prevalent one and the previous circulating one vanishes. In Figure 4 we consider the case where the initial number of infected people with the previously circulating strain is large in order to resemble reality when Omicron was introduced. It can be seen that despite having a large vaccination rate, the system (2) still approaches the endemic steady state $E_{1}^{*}$ due to the higher transmissibility of the Omicron strain. Figure 5 depicts the case where ${ℛ}_{0_{2}}>{ℛ}_{0_{1}}>1$ and it can be seen that the previously circulating strains and the Omicron strain become endemic. The explanation for this is due to the fact that people who got either of the SARS-CoV-2 strains can get the other strain. After this long-term dynamics results, the next subsection is devoted to the transient dynamics of the Omicron wave.

### 4.3. Numerical Simulations to Assess Critical Outcomes

For the in silico simulations we considered various efficacies of the vaccine against the Omicron strain, transmissibility and severity of the Omicron strain. In the analysis, we focus on the qualitative results and the effects of the aforementioned factors.

**Figure 6.**
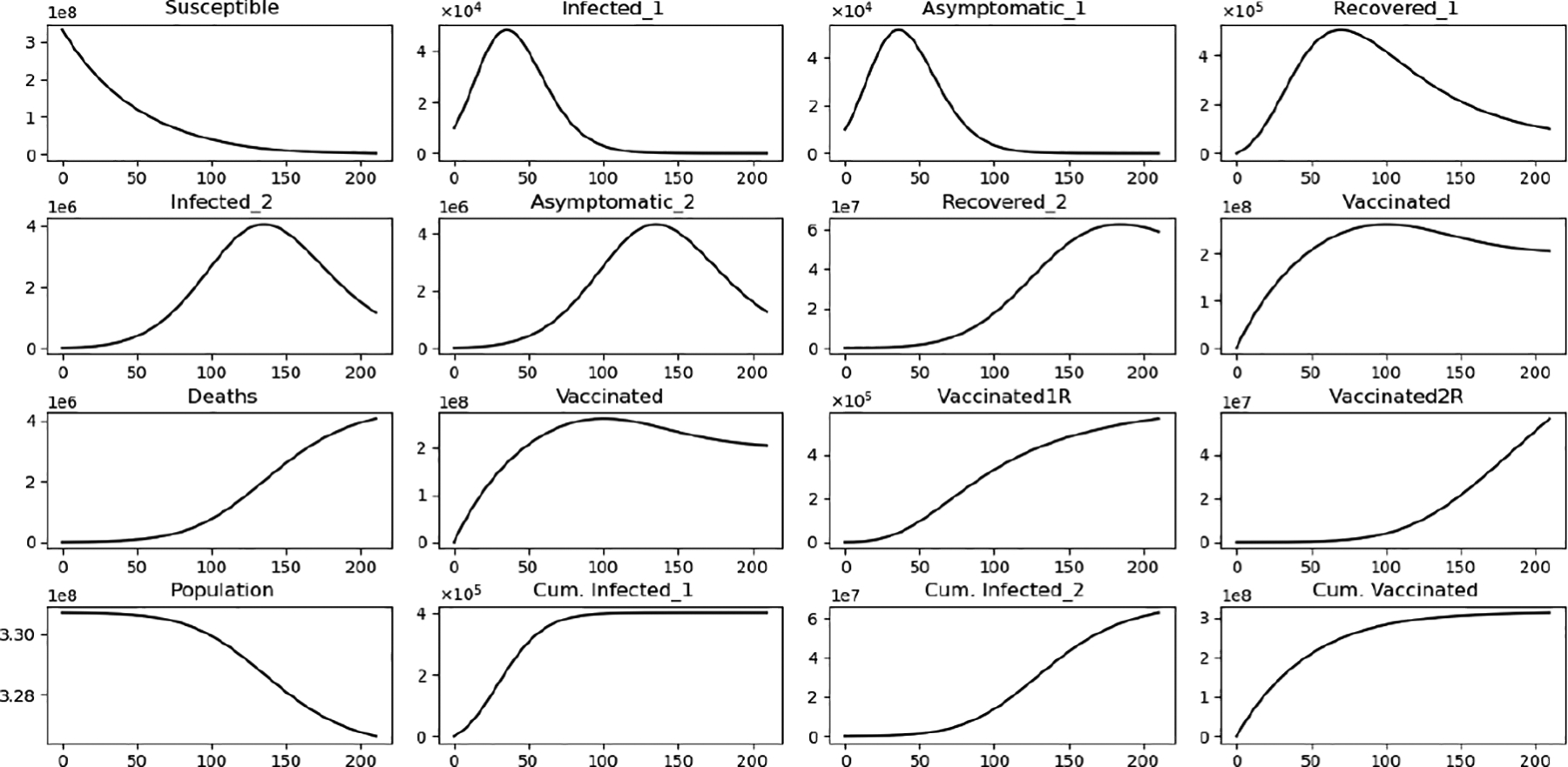
In silico simulation of the Omicron wave model (2) when ${ℛ}_{0_{2}}\approx 0.95>{ℛ}_{0_{1}}\approx 0.87$. The two strains vanish and the system approaches the disease-free equilibrium point $F_{1}^{*}$.

Figure 6 displays the paths of each of the subpopulations and some cumulative numbers. This is a particular case where we can see the evolution of the Omicron wave for one scenario. This is not a suitable way to understand the effects of the Omicron strain since there is no comparison with other scenarios. Thus, the next simulations consider variations of the vaccine’s efficacy against Omicron and also Omicron infectivity.

**Figure 7.**
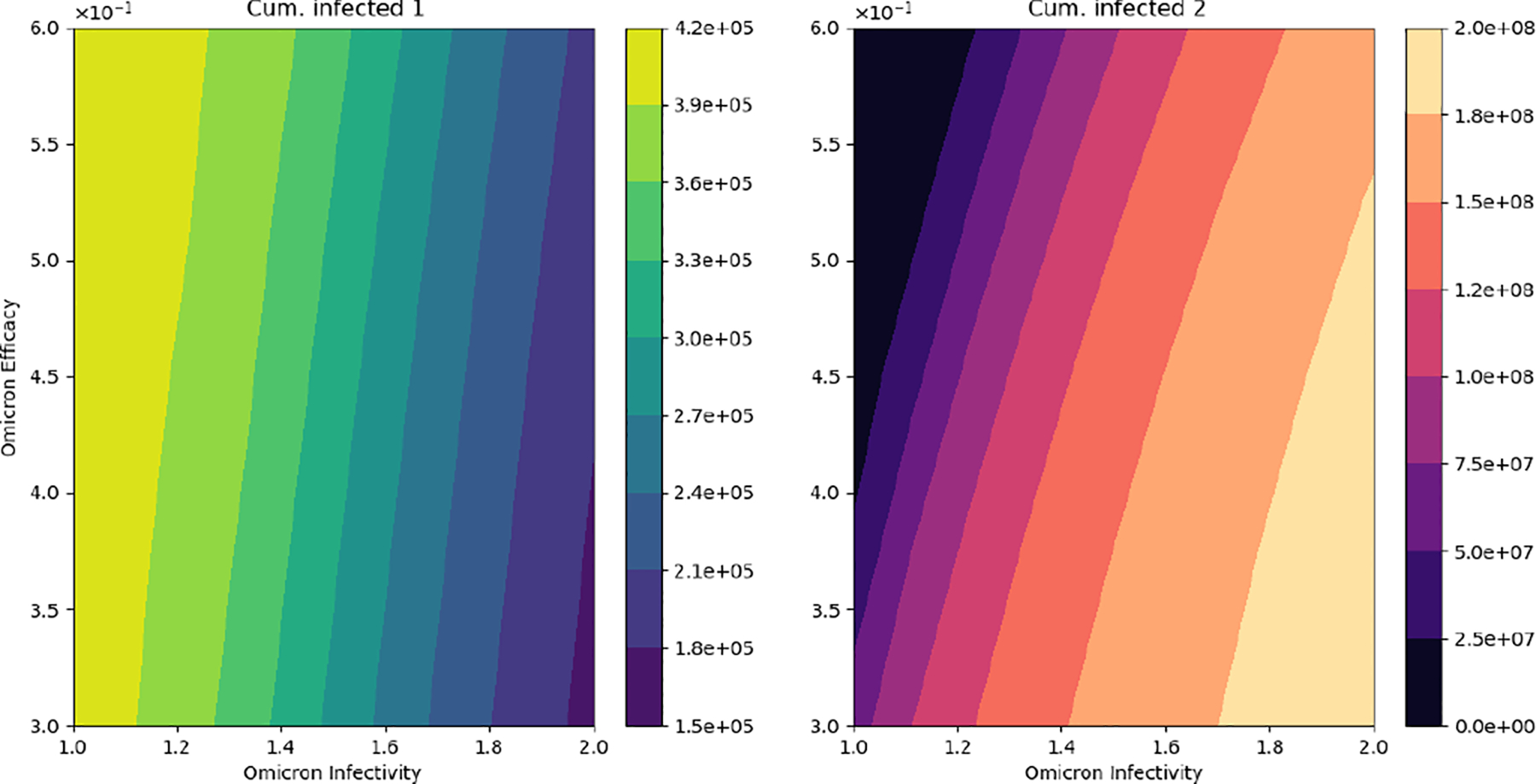
In silico simulation of the Omicron wave model (2). The outcomes regarding the final cumulative infected people for each strain. As the Omicron infectivity rate increases, the final cumulative number of people infected with Omicron increases but the final cumulative number of people infected with the previously circulating strain decreases.

Figure 7 displays different outcomes regarding the final cumulative infected population with each strain. It can be seen that when the Omicron infectivity rate increases, the final cumulative number of people infected with Omicron increases, but the final cumulative number of people infected with the previously circulating strain decreases. This is due to a competition for the susceptible people among the strains. The model does not consider co-infection. Furthermore, the final cumulative number of infected people with the previously circulating SARS-CoV-2 strain increases if the vaccine’s efficacy against Omicron increases. The opposite situation occurs for the final cumulative number of infected people with Omicron. However, the changes to final cumulative numbers for people infected with Omicron are much larger, which partially explains the large number of infected cases that have been recorded for the Omicron wave.

**Figure 8.**
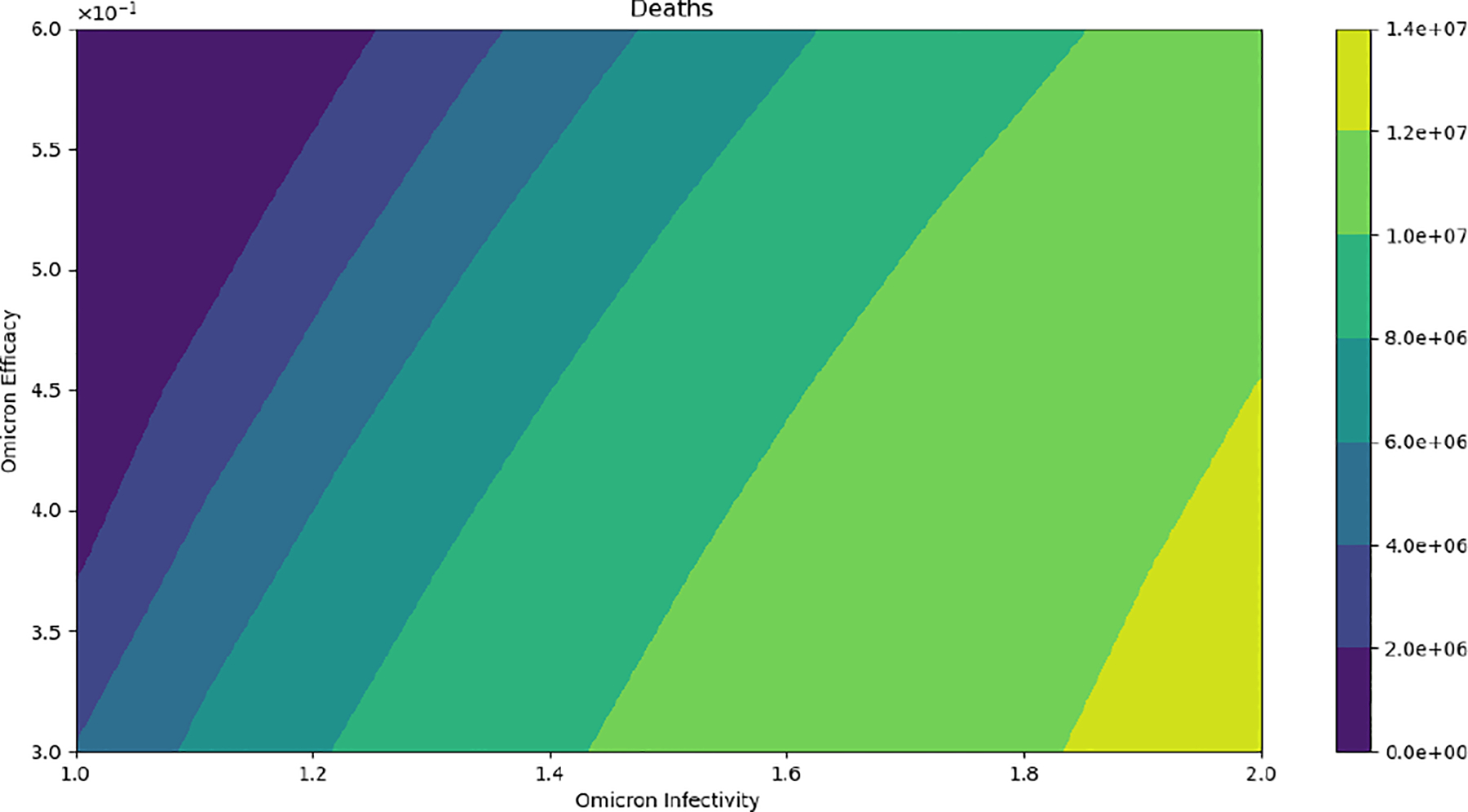
In silico simulation of the Omicron wave model (2). The outcomes regarding total deaths. As the Omicron infectivity rate increases, the final total number of deaths increases. As can be observed, the number of deaths increases despite assuming the same case fatality rate for the two strains.

Figure 8 displays the final cumulative number of deaths when we vary the vaccine’s efficacy against the Omicron strain and the infectivity of the Omicron strain. As can be observed, the final cumulative number of deaths increases as Omicron’s infectivity increases despite assuming the same case fatality rate. This is a major result to bring awareness to, given that even if the Omicron strain is less deadly the final cumulative deaths can increase as has indeed occurred [1,49]. We also performed in silico simulations assuming standard incidence in the model (2) and the results are qualitatively similar.

### 4.4. Comparison of the Omicron Wave with the Non-Omicron Situation

Finally, we present additional in silico simulations to compare the non-Omicron with the Omicron situation. In the analysis we focus on the qualitative results related to infected people and total number of deaths since these are the crucial health outcomes of the pandemic.

**Figure 9.**
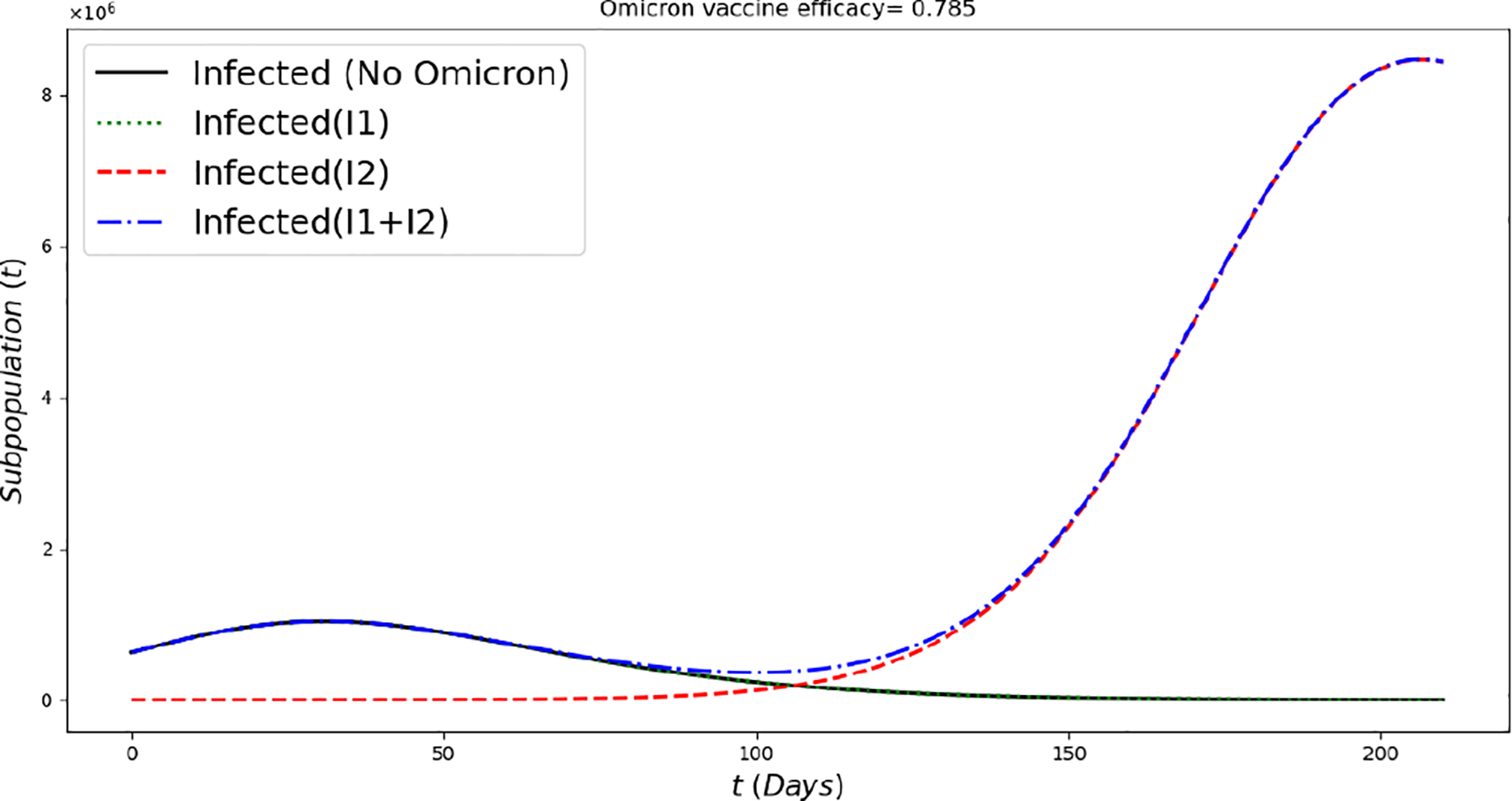
In silico simulation of the Omicron wave model (2) when ${ℛ}_{0_{1}}\approx 0.81,{ℛ}_{0_{2}}\approx 1.74$ and vaccine efficacy against Omicron is approximately 79%. More infected cases during the Omicron wave, despite a large number of the population being vaccinated and a relative acceptable vaccine efficacy.

Figure 9 displays the infected subpopulations over a period of six months. The total number of infected people is larger under the Omicron wave in comparison with the situation where no Omicron is introduced, as reflected in reality. Notice that initially the number of people infected with Omicron is much smaller, also as reflected in the real world.

**Figure 10.**
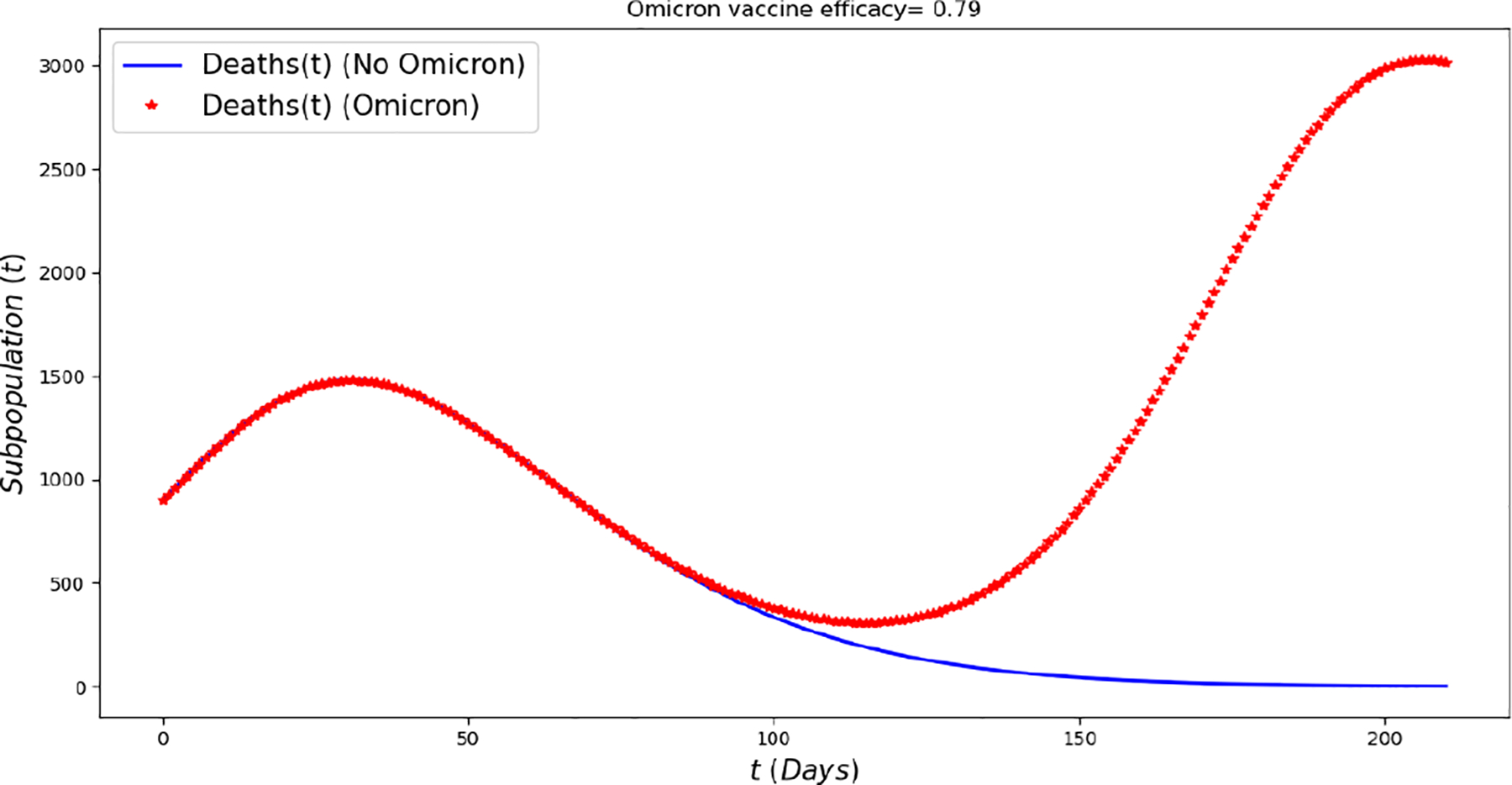
In silico simulation of the Omicron wave mathematical model (2) when ${ℛ}_{0_{1}}\approx 0.81,\;{ℛ}_{0_{2}}\approx 1.74$ and vaccine efficacy against Omicron is approximately 79%. More deaths during the Omicron wave, despite a lower case fatality rate for Omicron, large number of population vaccinated and a relative acceptable vaccine efficacy.

Figure 10 displays the number of deaths over a period of six months assuming a smaller death rate for people infected with Omicron (25% of previous circulating strain). The total number of deaths is larger under the Omicron wave in comparison with the situation with no Omicron despite a large number of the population being vaccinated and a relative acceptable vaccine efficacy. These results are in good agreement with the results that have occurred during the Omicron wave [1,49].

### 4.5. Discussion of Numerical Simulation Results

The numerical simulation results presented here agree with those obtained in previous work related to the Omicron wave. For instance, in [121] the authors found that a new SARS-CoV-2 variant’s (for example, Omicron) dominance is primarily driven by its infectivity, which does not always lead to an increased public health burden. This has been shown in our work through the theoretical results and the numerical simulations. In [119], the authors considered several key uncertainties and concluded that in the particular case of England and under the assumption that no new variants emerge, SARS-CoV-2 transmission is expected to decline. This also agrees with our results, since the basic effective reproductive number depends on the transmission rates. The authors mentioned that the projections depend largely on assumptions around waning immunity, social behavior and seasonality. In our work, we presented sensitivity analysis to assess the effects of uncertainty of some factors related to the Omicron variant and the results agree with the aforementioned work. It is important to remark that during the Omicron wave, people from each region have different levels of immunity protection. This was investigated in in [120]. Their results suggested that even in those regions where the Delta variant is controlled before the beginning of the Omicron wave a significant Omicron wave can be expected. This has been shown in our study and under some mathematical conditions that we have found. Thus, all these results provide additional insight into the understanding of new SARS-CoV-2 variants.

Previous studies have modeled the Omicron wave [119–121]. In [121], the authors implemented a stochastic, discrete-time- and individual-based transmission model of SARS-CoV-2 infection and COVID-19 disease. The model considers an age-structured, small-world network. Using sensitivity analysis, they show that a new SARS-CoV-2 variant dominance is primarily driven by its infectivity, which does not necessarily lead to an increased public health burden. In [119] the authors used a model fitted to more than 2 years of epidemiological data from England to project potential dynamics of SARS-CoV-2 infections and deaths to December 2022. They considered several key uncertainties including behavioral change and waning immunity. They concluded that for the particular case of England and under the assumption that no new variants emerge, SARS-CoV-2 transmission is expected to decline. The authors concluded that the projections depend largely on assumptions of waning immunity, social behavior and seasonality. Other interesting work related to Omicron waves is presented in [120]. In this work, a generalized SEIR model assuming gamma-distributed incubation and infectious periods is presented. The model includes susceptibility to Omicron. Their results suggest that even in those regions where the Delta variant is controlled before the beginning of the Omicron wave a significant Omicron wave can be expected. For the particular case of England, the Omicron wave was smaller than the Delta wave. In our paper, we provide additional insight regarding the Omicron wave.

## 5. Conclusions

Mathematical models are fruitful for the study of various epidemics and infectious diseases. The models allow us to learn about the evolution of epidemics and also to grasp the potential effects of public health control strategies on the epidemics. Forecasting epidemics is frequently a complex task. Mathematical models are able to provide results that sometimes are difficult to anticipate without mathematical tools.

We constructed a mathematical model to investigate the evolution of the Omicron wave. The Omicron strain has caused a new wave with a large amount of infected cases and deaths worldwide. In some countries, the average number of deaths during this Omicron wave has only slightly increased in comparison with previous circulating SARS-CoV-2 waves. We used a mathematical model to study and approximate the Omicron wave situation in the USA, but it can be extended to other countries. This study uses the facts that the Omicron strain exhibits a higher intrinsic transmissibility than the previously circulating SARS-CoV-2 strain but is less deadly. The numerical simulation results show that despite the fact that the Omicron strain is less deadly it can nevertheless cause more deaths and hospitalizations. This result is of paramount importance for public health, since many people might think that since the Omicron strain is less deadly then the number of deaths will be fewer during the Omicron wave. The spread of the Omicron strain depends on several factors, which vary according to the region; therefore, the Omicron wave situation can be different in other countries or regions. In summary, we used a mathematical model in conjunction with numerical simulations to provide an explanation of a large Omicron wave under various conditions related to the variant’s transmissibility. These results provide awareness that new SARS-CoV-2 variants can cause more deaths even if their fatality rate is lower. In fact, we can mention that in the USA the peak of number of deaths during the Omicron wave was comparable to that during the Delta wave despite the fact that during the former wave people already had immunity protection due to vaccination programs [1]. In addition, in Brazil and Colombia, the numbers of infected cases were larger than those during the Delta wave. These facts point out the different potential outcomes of new SARS-CoV-2 variants with different transmissibility and fatality rates.

From a mathematical analysis viewpoint, we studied first the local stability using the well-known NGM method. We computed the basic reproduction number ${ℛ}_{0}$ and found that it is the largest of the two parameters ${ℛ}_{0_{1}}$ and ${ℛ}_{0_{2}}$. This theoretical result reveals that the COVID-19 pandemic can become extinct if ${ℛ}_{0}<1$. This is achievable if the vaccination rate is increased (this implies that people are willing to get vaccinated) and/or the transmission rate is decreased such that ${ℛ}_{0}<1$. We also performed global stability analysis for the disease-free steady state. The numerical simulations provided additional support to the theoretical analysis and showed qualitative effects of the Omicron strain on the US population. This study is more designed for a relatively short time horizon. However, we provide long-term mathematical analysis to obtain a better picture of the dynamics. Interesting and deeper mathematical analysis can be be carried out regarding the endemic states, global stability, periodic solutions and bifurcations.

We provided a variety of scenarios that help to obtain insight into the Omicron wave and its consequences. The numerical simulations showed the Omicron wave outcomes under different conditions related to the vaccines and transmissibility. The results show that the final cumulative number of infected people can be greater with respect to previous waves despite a large number of people being vaccinated. These results are in good agreement with what has occurred during the Omicron wave. For instance, this happened in Brazil and Colombia [1,49].

The results presented here help to support public health policies and, most importantly, to bring awareness to people about the Omicron strain or future highly contagious SARS-CoV-2 strains. At this time, China is suffering one of the largest waves in spite of the fact that in the past they were able to control the spread of SARS-CoV-2. As in any mathematical model, we need to be aware of the limitations in order to understand potential misunderstandings or mistaken conclusions. For instance, the constructed mathematical model assumes homogeneous mixing and constant proportional vaccination rates which obviously is not the case in the real world. One way to better approximate reality would be to describe the vaccination using real data which would give a more complex model since it would then become non-autonomous (see [105]). In addition, more detailed models can include age structure and seasonality. However, despite the usual limitations of mathematical models, this study provides useful means of explaining and obtaining deeper insight about the Omicron wave. As shown by the simulations, the appearance of the Omicron strain or highly contagious SARS-CoV-2 strains changes the dynamics of the pandemic and can increase the number of deaths despite a lower mortality rate.

As in any mathematical model of the real world there are limitations in the results and conclusions. The proposed model is just an approximation of the reality during the Omicron wave. During this wave several SARS-CoV-2 variants were circulating. The model assumes the existence of just two main variants. The model assumes a constant transmission rate for each of the Delta and Omicron variants, but the reality is that these rates change continuously depending on many complex factors. The proposed model does not consider explicitly people hesitant to be vaccinated. The model does not consider the spatial effects of the diffusion of SARS-CoV-2. This has been a common weakness of many models. The model considers only one vaccinated population without any distinction between the number of doses received by individuals. The model does not include human behavioral changes, but considers a variety of transmission rates in the sensitivity analysis.

Finally, we would like to point out that the results presented here are helpful to obtain further insight into the Omicron wave and the effect of new highly transmissible strains and new vaccines. Various graphical illustrations show the impact of vaccines and transmissibility on the Omicron wave. From the results, it can be seen that the COVID-19 pandemic can be eliminated under some circumstances and following the recommendations of the World Health Organization (WHO).

## Figures and Tables

**Table 1.** Parameters for the Omicron wave mathematical model (2) with their respective meaning and numerical values.

Parameter	Symbol	Value
Inflow rate	λ	7.864180 × 10^3^ people/day [130]
Natural death rate	d	0.00002378 1/day [130]
Infectious period	$\gamma^{-1}$	7 days [131]
Transmission rate	$\beta_{i}$	varied $\beta_{1}\leq \beta_{2}$
Death rate (infected with previous circulating strains)	$d_{1}$	0.01 days^−1^ [106,132]
Death rate (infected with Omicron)	$d_{2}$	varied < 0.01 days^−1^ [49]
Vaccination rates	$\nu ,v_{R}$	varied $v\geq v_{R}$ 1/day [130]
Proportion of asymptomatic	$a_{i}$	0.5 [133,134]

**Table 2.** Values of the assumed efficacies for the SARS-CoV-2 vaccines used in the in silico simulations.

Parameter	From	To	Value
$\epsilon_{1}$	V	$I_{1},A_{1}$	[0.8,0.95]
$\epsilon_{1}$	$R_{1}$	$I_{1},A_{1}$	[0.8,0.95]
$\epsilon_{2}$	V	$I_{2},A_{2}$	[0.37,0.6]
$\epsilon_{1R}$	$V_{1R}$	$I_{1},A_{1}$	[0.98,0.99]
$\epsilon_{12R}$	$V_{1R}$	$I_{2},A_{2}$	[0.95,0.98]
$\epsilon_{22}$	$R_{2}$	$I_{2},A_{2}$	[0.9,0.95]
$\epsilon_{21}$	$R_{2}$	$I_{1},A_{1}$	[0.37,0.6]
$\epsilon_{21R}$	$V_{2R}$	$I_{1},A_{1}$	[0.98,0.99]

## Data Availability

Data are contained within the article. Codes are available upon request.
